# Advanced MR Techniques for Preoperative Glioma Characterization: Part 2

**DOI:** 10.1002/jmri.28663

**Published:** 2023-03-13

**Authors:** Gilbert Hangel, Bárbara Schmitz‐Abecassis, Nico Sollmann, Joana Pinto, Fatemehsadat Arzanforoosh, Frederik Barkhof, Thomas Booth, Marta Calvo‐Imirizaldu, Guilherme Cassia, Marek Chmelik, Patricia Clement, Ece Ercan, Maria A. Fernández‐Seara, Julia Furtner, Elies Fuster‐Garcia, Matthew Grech‐Sollars, N. Tugay Guven, Gokce Hale Hatay, Golestan Karami, Vera C. Keil, Mina Kim, Johan A. F. Koekkoek, Simran Kukran, Laura Mancini, Ruben Emanuel Nechifor, Alpay Özcan, Esin Ozturk‐Isik, Senol Piskin, Kathleen M. Schmainda, Siri F. Svensson, Chih‐Hsien Tseng, Saritha Unnikrishnan, Frans Vos, Esther Warnert, Moss Y. Zhao, Radim Jancalek, Teresa Nunes, Lydiane Hirschler, Marion Smits, Jan Petr, Kyrre E. Emblem

**Affiliations:** ^1^ Department of Neurosurgery Medical University of Vienna Vienna Austria; ^2^ High Field MR Centre, Department of Biomedical Imaging and Image‐guided Therapy Medical University of Vienna Vienna Austria; ^3^ Christian Doppler Laboratory for MR Imaging Biomarkers Vienna Austria; ^4^ Medical Imaging Cluster Medical University of Vienna Vienna Austria; ^5^ Department of Radiology Leiden University Medical Center Leiden the Netherlands; ^6^ Medical Delta Foundation Delft the Netherlands; ^7^ Department of Diagnostic and Interventional Radiology University Hospital Ulm Ulm Germany; ^8^ Department of Diagnostic and Interventional Neuroradiology, School of Medicine, Klinikum rechts der Isar Technical University of Munich Munich Germany; ^9^ TUM‐Neuroimaging Center, Klinikum rechts der Isar Technical University of Munich Munich Germany; ^10^ Institute of Biomedical Engineering, Department of Engineering Science University of Oxford Oxford UK; ^11^ Department of Radiology & Nuclear Medicine Erasmus MC Rotterdam Netherlands; ^12^ Department of Radiology & Nuclear Medicine Amsterdam UMC, Vrije Universiteit Amsterdam Netherlands; ^13^ Queen Square Institute of Neurology and Centre for Medical Image Computing University College London London UK; ^14^ School of Biomedical Engineering and Imaging Sciences King's College London London UK; ^15^ Department of Neuroradiology King's College Hospital NHS Foundation Trust London UK; ^16^ Department of Radiology Clínica Universidad de Navarra Pamplona Spain; ^17^ Rede D'Or São Luiz Hospital Santa Luzia Brazil; ^18^ Department of Technical Disciplines in Medicine, Faculty of Health Care University of Prešov Prešov Slovakia; ^19^ Department of Diagnostic Sciences Ghent University Ghent Belgium; ^20^ Department of Medical Imaging Ghent University Hospital Ghent Belgium; ^21^ IdiSNA, Instituto de Investigación Sanitaria de Navarra Pamplona Spain; ^22^ Department of Biomedical Imaging and Image‐guided Therapy Medical University of Vienna Vienna Austria; ^23^ Research Center of Medical Image Analysis and Artificial Intelligence Danube Private University Austria; ^24^ Biomedical Data Science Laboratory, Instituto Universitario de Tecnologías de la Información y Comunicaciones Universitat Politècnica de València Valencia Spain; ^25^ Centre for Medical Image Computing, Department of Computer Science University College London London UK; ^26^ Lysholm Department of Neuroradiology, National Hospital for Neurology and Neurosurgery University College London Hospitals NHS Foundation Trust London UK; ^27^ Institute of Biomedical Engineering Bogazici University Istanbul Istanbul Turkey; ^28^ Cancer Center Amsterdam Amsterdam Netherlands; ^29^ Centre for Medical Image Computing, Department of Medical Physics & Biomedical Engineering and Department of Neuroinflammation University College London London UK; ^30^ Department of Neurology Leiden University Medical Center Leiden the Netherlands; ^31^ Department of Neurology Haaglanden Medical Center Netherlands; ^32^ Department of Bioengineering Imperial College London London UK; ^33^ Department of Radiotherapy and Imaging Institute of Cancer Research UK; ^34^ Department of Brain Repair and Rehabilitation, Institute of Neurology University College London London UK; ^35^ Department of Clinical Psychology and Psychotherapy, International Institute for the Advanced Studies of Psychotherapy and Applied Mental Health Babes‐Bolyai University Romania; ^36^ Electrical and Electronics Engineering Department Bogazici University Istanbul Istanbul Turkey; ^37^ Department of Mechanical Engineering, Faculty of Natural Sciences and Engineering Istinye University Istanbul Istanbul Turkey; ^38^ Department of Biophysics Medical College of Wisconsin Milwaukee Wisconsin USA; ^39^ Department of Physics and Computational Radiology Oslo University Hospital Oslo Norway; ^40^ Department of Physics University of Oslo Oslo Norway; ^41^ Department of Imaging Physics Delft University of Technology Delft the Netherlands; ^42^ Faculty of Engineering and Design Atlantic Technological University (ATU) Sligo Sligo Ireland; ^43^ Mathematical Modelling and Intelligent Systems for Health and Environment (MISHE), ATU Sligo Sligo Ireland; ^44^ Department of Radiology Stanford University Stanford California USA; ^45^ Stanford Cardiovascular Institute Stanford University Stanford California USA; ^46^ Department of Neurosurgery St. Anne's University Hospital Brno Czechia; ^47^ Faculty of Medicine Masaryk University Brno Czechia; ^48^ Department of Neuroradiology Hospital Garcia de Orta Almada Portugal; ^49^ C.J. Gorter MRI Center, Department of Radiology Leiden University Medical Center Leiden the Netherlands; ^50^ Brain Tumour Centre Erasmus MC Cancer Institute Rotterdam the Netherlands; ^51^ Helmholtz‐Zentrum Dresden‐Rossendorf Institute of Radiopharmaceutical Cancer Research Dresden Germany

**Keywords:** glioma, brain, preoperative, contrasts, GliMR 2.0, level of clinical validation

## Abstract

Preoperative clinical MRI protocols for gliomas, brain tumors with dismal outcomes due to their infiltrative properties, still rely on conventional structural MRI, which does not deliver information on tumor genotype and is limited in the delineation of diffuse gliomas. The GliMR COST action wants to raise awareness about the state of the art of advanced MRI techniques in gliomas and their possible clinical translation. This review describes current methods, limits, and applications of advanced MRI for the preoperative assessment of glioma, summarizing the level of clinical validation of different techniques. In this second part, we review magnetic resonance spectroscopy (MRS), chemical exchange saturation transfer (CEST), susceptibility‐weighted imaging (SWI), MRI‐PET, MR elastography (MRE), and MR‐based radiomics applications. The first part of this review addresses dynamic susceptibility contrast (DSC) and dynamic contrast‐enhanced (DCE) MRI, arterial spin labeling (ASL), diffusion‐weighted MRI, vessel imaging, and magnetic resonance fingerprinting (MRF).

**Evidence Level:**

3.

**Technical Efficacy:**

Stage 2.

Gliomas are a heterogeneous group of primary brain tumors that arise from the glial cells, with a dismal prognosis despite standard‐of‐care oncological treatment.^1^ While conventional structural MRI with T1‐ and T2‐weighted, fluid‐attenuated inversion recovery (FLAIR) sequences, and gadolinium‐based contrast agents (GBCA) is routinely applied in the preoperative workup of patients with glioma,^2^ advanced MRI may provide additional opportunities to map tumor features, facilitate noninvasive genotyping, and optimize treatment strategies.^3^

To support the clinical use of advanced MRI, we have reviewed current advanced MRI techniques and scored their level of clinical validation and hence technology readiness in the context of preoperative glioma imaging. The first part of this review includes perfusion imaging by dynamic contrast‐enhanced (DCE), dynamic susceptibility contrast (DSC), and arterial spin labeling (ASL), as well as diffusion MRI, vessel imaging, and relaxometry and MR fingerprinting (MRF).

This second part focuses on metabolic and chemical‐composition imaging with MR spectroscopy (MRS) and chemical exchange saturation transfer (CEST), susceptibility‐weighted imaging (SWI), MR elastography (MRE), and combined imaging by MRI and positron emission tomography (MRI‐PET). Finally, we discuss the potential clinical use of advanced imaging biomarkers for glioma characterization in the context of radiomics and deep learning.

## Methods

This review was initiated through the European Cooperation in Science and Technology (COST) Glioma MR Imaging 2.0 (GliMR) initiative.^4^ We aimed to use the GliMR consortium's technical and clinical expertise to aggregate the available evidence and the level of clinical and technological validation for cutting‐edge MRI methods and the information derivable from them (Table 1). For the sake of consistency with previously published reviews by GliMR, with consent from the authors, we adopted the format used in the study by Booth et al.^5^

Detailed methods are described in the first part of this review [note: reference during layout].

**TABLE 1:** Level of Validation Table

	Track and Domain^a^	MRS		CEST	T2*	PET	MRE	Criteria			
		Single	CSI	APT	SWI	AA		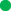	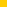	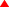	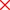
Technical validation											
Test–retest repeatability	T2	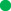	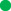	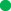	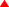	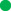	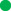	Yes, with current standard implementation	Yes, but with other implementation or patient group/animal model	None available	Unclear
Cross‐vendor reproducibility	T2	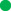	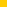	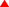	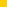	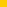	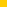	Yes, with current standard implementation	Yes, but with other implementation or patient group	None available	Unclear
Multisite reproducibility	T3	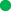	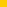	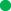	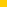	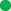	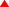	Yes, with current standard implementation	Yes, but with other implementation, or patient group, phantom, or analysis	None available	Unclear
Clinical evidence											
Proof‐of‐concept in patients	C1	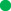	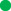	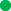	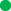	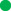	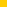	Differentiation of tumor types/grades	Differentiation of tumor from normal brain	None available	Unclear
Evaluated in clinical studies	C2‐3	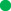	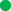	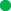	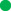	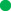	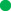	Multiple single center	Few or preliminary studies	None available	Unclear
Evaluated in multicenter studies	C3	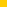	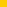	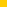	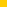	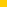	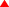	Good quality with relevant question	Small, preliminary or only method stability/not relevant question	None available	Unclear
Evaluated in meta‐analysis		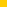	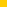	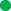	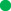	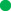	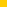	Consistent result with standard measure	No standard measure/method, or low number of studies/patients	None available	Unclear
Established diagnostic accuracy, cut‐offs/criteria	C3	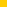	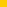	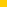	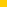	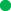	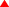	Consistent in multiple single‐center studies	Few or preliminary studies	None available	Unclear
Acceptance											
Method guidelines recommendations	T	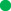	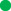	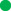	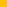	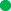	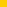	Available and updated	Available, but not updated or not specific for tumor imaging	None available	Unclear
Included in national imaging guidelines		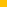	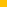	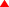	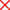	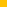	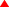	Endorsed by a majority of the community	Only endorsed by a minority	Not mentioned	Unclear
Included in clinical trial guidelines^b^		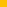	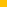	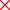	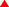	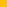	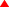	Included in suggested standard protocol	Mentioned, but clinical value uncertain	Not mentioned	Unclear
Included in international clinical guidelines^c^		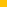	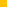	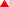	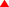	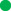	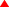	Endorsed by major international society guidelines	Mentioned, but clinical value uncertain	Not mentioned	Unclear
In clinical use for brain tumor imaging^d^		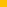	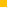	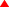	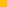	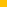	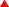	Widely implemented (>50%)	Intermediate (<50%)	Uncommon	Unclear
In clinical use for glioma diagnosis^d^		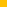	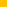	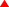	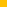	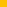	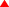	Widely applied (>50%)	Intermediate (<50%)	Uncommon	Unclear
Implementation											
Sequence availability	T2	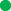	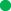	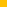	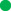	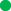	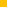	Comparable sequence available as clinical from all major vendors	No standard implementation or only WIP	Research sequence at single sites	Unclear
Postprocessing software availability	T2	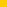	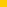	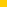	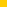	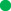	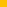	On‐line scanner/reading workstation with best‐practice implementation	Off‐line, commercially available software	In‐house software	Unclear
Subjective ease of data acquisition (scanner operator)	T2	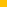	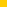	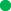	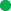	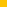	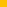	Minimal need for training	Special training/attention required	Difficult to obtain good quality data	Unclear
Subjective ease of postprocessing (within clinical department)		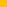	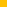	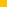	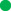	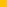	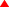	No postprocessing needed	Extra processing/training needed, but not time‐consuming	Expert or time‐intensive processing required	Unclear
Subjective ease of data interpretation (clinician)		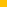	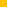	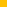	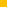	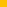	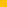	Visual reading or only simple manual steps required	Special training/expertise required	Highly specialized in single centers	Unclear

References to the guidelines and also further material for each technique are included in the supplementary materials S1.

T = technical validation; C = clinical validation; Domain 1 = discovery; Domain 2 = validation; Domain 3 = Qualification.

^a^ Imaging biomarker roadmap.

^b^ RANO, iRANO, Standardized Brain Tumor Imaging Protocol.

^c^ GBM EANO/SNO, EANO diff. glioma, EANO glioma.

^d^ European survey of advanced MRI, US survey of perfusion imaging.

## Results

### MR Spectroscopy

#### OVERVIEW

MR spectroscopy (MRS) techniques allow the noninvasive detection and quantification of tissue metabolites that differ in their resonance frequency profiles by a few parts per million. Since these molecules are many times less abundant than water, applications focus on either single‐voxel spectroscopy (SVS) or multivoxel MRS imaging (MRSI) with a much larger voxel size than conventional MRI. However, most applications focus on point‐resolved spectroscopy (PRESS).^6^ The most commonly used MRS technique in the clinical setting is proton MRS (1H‐MRS), which can detect many metabolites, including *N*‐acetyl‐aspartate (NAA), creatine (Cr), choline (Cho), myo‐inositol (mI), glutamate (Glu), glutamine (Gln), gamma‐aminobutyric acid (GABA), glutathione (GSH), lactate (Lac), lipid (Lip), and also the recently described oncometabolite 2‐hydroxyglutarate (2HG).^7^, ^8^ While 2HG is a specific, and thus, a highly valuable marker of IDH‐mutated gliomas, quantifying anything other than the ratio of Cho/NAA is difficult in a clinical setup without MRS experts, as decisions on acquisition, signal processing, and fitting of resonance peaks affect the results to a greater degree than in conventional MRI.

#### CLINICAL APPLICATION

##### Proton magnetic resonance spectroscopy

**FIGURE 1:**
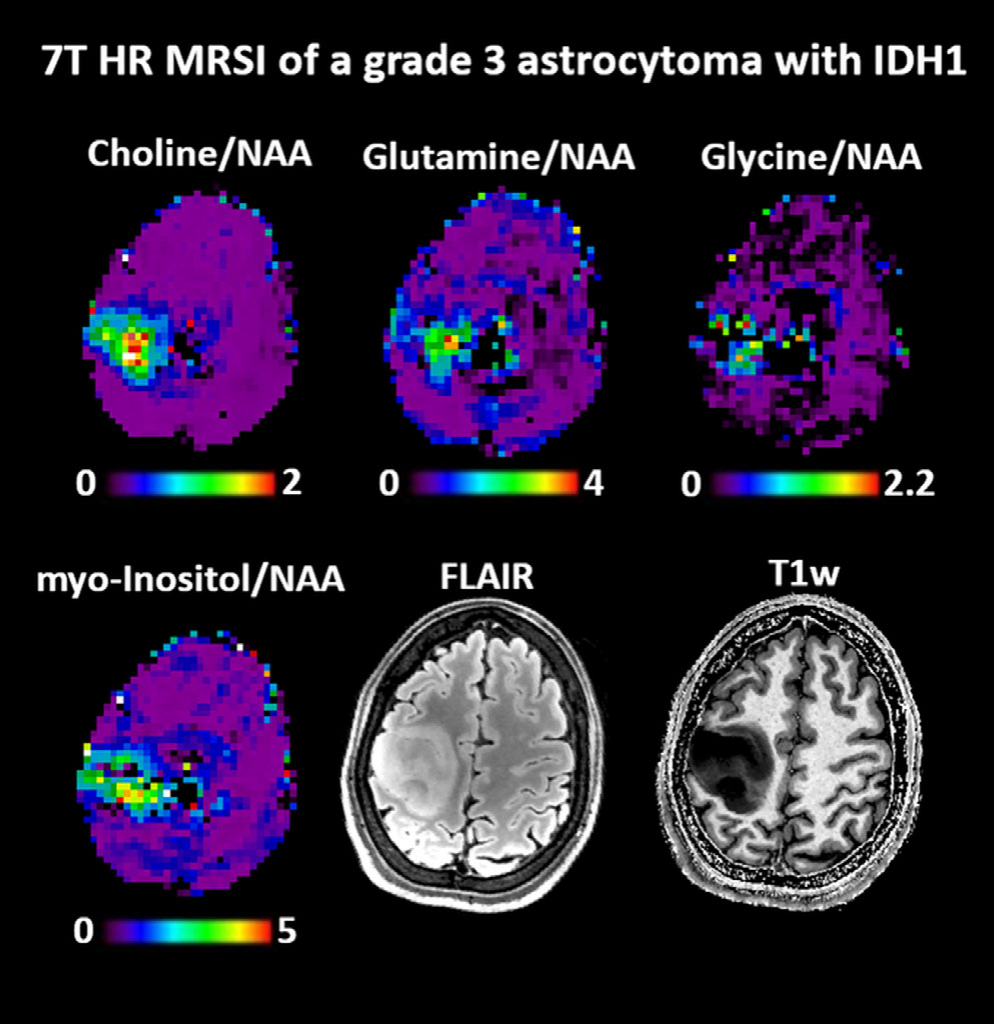
Metabolic ratio maps of a WHO grade 3 astrocytoma with IDH mutation (2016 WHO classification) obtained with a 7 T MRSI method that acquired 3D metabolic images with 3.4 mm nominal resolution in 15 minutes. All displayed oncometabolites to NAA are dominantly increased, but also show possible heterogeneities in the glioma metabolism.^9^

1H‐MRS may aid in distinguishing gliomas from conditions that may mimic gliomas in conventional MRI, including non‐neoplastic (such as tumefactive demyelinating lesions) and neoplastic lesions (namely metastasis and primary CNS lymphoma). It could also help differentiate between edema, gliosis, and infiltrative tumor tissue in heterogeneous glioma and could also be applied as 1H‐MRSI (Fig. 1).^9^, ^10^

**FIGURE 2:**
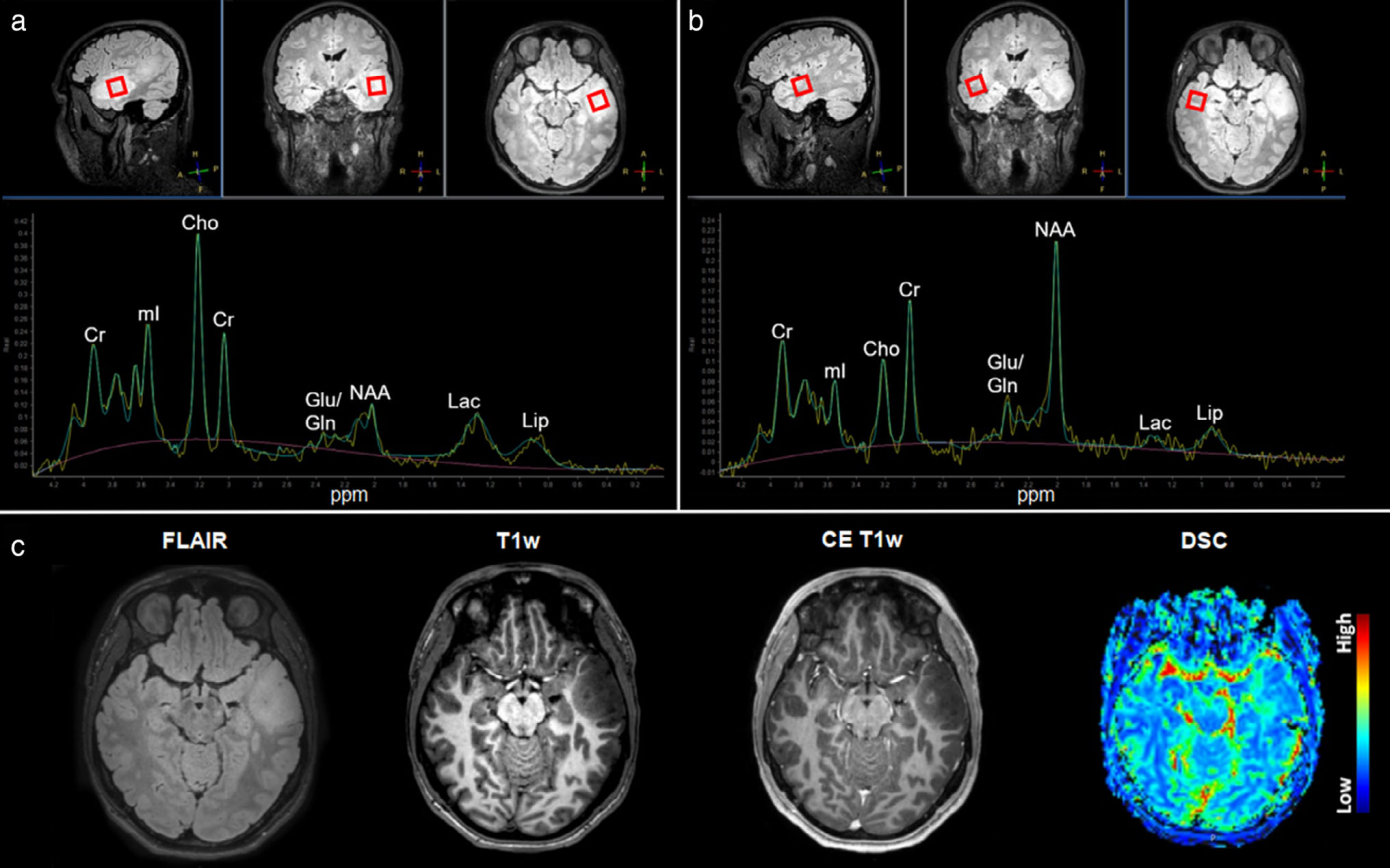
A 3 T 1H‐MRS from a 35‐year‐old male with a left temporal lesion. (a) The voxel for measurement placed in the FLAIR‐hyperintense lesion of the left anterior temporal lobe (red box) together with the measured and fitted spectrum. (b) The voxel for measurement placed in the contralateral healthy side (red box) with the resulting measured and fitted spectrum from the same patient. The spectrum depicts major metabolites resonating at typical ppm (x‐axis). Measurements from the lesion showed elevated Cho (inversion of the Cho/Cr ratio), decreased NAA, and a modest Lac increase when compared to the contralateral measurements. (c) Corresponding axial slices from FLAIR, noncontrast T1‐, contrast‐enhanced T1‐weighted, and DSC perfusion imaging, characterizing the lesion as FLAIR‐hyperintense with discrete focal contrast enhancement and related focal hyperperfusion. The histopathological diagnosis after the biopsy was glioblastoma.

Most previous 1H‐MRS/I glioma studies indicated changes in the levels of Cho, Cr, NAA, Lac, and Lip compared to healthy tissue (Fig. 2). Common observations were increases in Cho levels due to elevated cell density and/or membrane turnover in neoplasms, in addition to decreases in NAA levels, all of which are suggestive of axonal degeneration or loss. In addition, a relative decrease in Cr and increased levels of Lac and Lip were observed in glioblastoma.^7^ While both gliomas and metastases show increased Cho, lipids and macromolecules are higher in metastases than in glioblastoma. Due to the infiltrative nature of gliomas, spectroscopic assessment of edema next to the enhancing mass may be particularly useful, showing higher Cho/NAA and Cho/Cr in gliomas compared to metastases. Although both glioblastoma and primary CNS lymphoma show Cho/NAA elevation, in lymphomas, this ratio is reported to be lower.

Spectroscopy may aid in the differentiation of lower from HGGs using the Cho/Cr, NAA/Cr, and especially Cho/NAA ratios,^11^ despite a considerable variation of the ration cut‐offs. Moreover, 1H‐MRS has been suggested for the differentiation between high‐ and low‐grade oligodendrogliomas.^12^

Furthermore, glioma with mutations in the isocitrate dehydrogenase (IDH) enzyme (IDH‐mutant), which could cause accumulation of the oncometabolite 2HG, has been the focus of recent research using 1H‐MRS/I. A recent review estimated that the pooled sensitivity and specificity of 2HG diagnostic performance in IDH‐mutant glioma prediction was 95% and 91%, respectively.^13^ Furthermore, in cases with unclear IDH‐mutant status, 2HG has increased the correct diagnosis rate.^14^ In addition to elevated 2HG, IDH‐mutant tumors have displayed lower nicotinamide adenine dinucleotide phosphate (NADPH), GSH, Glu, and Gln, and higher mI and NAA levels compared to IDH wild‐type tumors. Together, these metabolic markers have been used to identify IDH‐mutant gliomas with over 88% accuracy.^15^ Another negative prognostic factor is the telomerase reverse transcriptase promoter (TERTp) mutation. IDH wild‐type gliomas that are TERTp mutant (TERTp‐only) have been reported to have the worst overall survival despite the tumor grade.^16^ A recent study indicated that TERTp‐only gliomas could be identified with a high accuracy of 92.6% based on total Cho and Glu and Gln complex (Glx) levels.^15^

Despite the promising role of spectroscopy, there is considerable overlap of spectra in different conditions, stressing the benefit of spectroscopy to be interpreted in the context of conventional imaging findings.

##### Carbon and Phosphorus Magnetic Resonance Spectroscopy

Hyperpolarized 13C‐MRS may be used to monitor metabolic fluxes. Specifically, [1‐13C] α‐ketoglutarate can be an imaging agent for real‐time in vivo monitoring of IDH‐mutant activity via the accumulation of [1‐13C]‐2‐HG, as demonstrated in vivo and in an orthotopic preclinical model engineered to express IDH mutant.^17^ Moreover, a drop in the conversion of [1‐13C] α‐ketoglutarate to [1‐13C]‐Glu was also observed in the same orthotopic glioma model, which was correlated with a drop in the activity and expression of several enzymes (BCAT1, ASTI1/2, GDH1/2) that catalyze the α‐ketoglutarate‐to‐Glu conversion, which is related to their 2HG‐induced promoter methylation and silencing.^17^

Phosphorus MRSI (31P‐MRSI) provides quantitative information about the energetic and ischemic state, membrane degradation and synthesis, and pH of the tissue of interest.^18^ In brain tumors, a decrease in phosphocreatine (PCr) and increases in inorganic phosphate (Pi), phosphocholine (PC), phosphoethanolamine (PE), glycerophosphocholine (GPC), and glycerophosphoethanolamine (GPE) have been reported.^18^, ^19^ In addition, a previous study indicated that pH, as well as the phosphomonoester over phosphodiester ratio (PME/PDE), and the PDE/Pi, PME/PCr, and PDE/PCr ratios could be used to distinguish between different types of brain tumors.^20^

#### VALIDATION

Many of the drawbacks of 1H‐MRS/I, especially with regard to quantification and artifacts, can be handled with some software improvements on current scanner hardware.^21^ The lack of consistent 1H‐MRS application guidelines has resulted in the technique being regarded as investigational rather than clinical, even after several years of application.^7^ Recent efforts toward consensus recommendations for MRS data acquisition are expected to help with this issue,^10^ although there are still no uniformly accepted thresholds for specific indications in neuro‐oncology.^3^

From a practical point of view, relatively long data acquisition times, challenging voxel/slab placement, and a low signal‐to‐noise ratio (SNR) still hamper the feasibility of 1H‐MRS/I in clinical settings.^22^ The wider availability of higher field strength scanners and phased array coils have improved the SNR for MRS. Parallel imaging techniques have enabled faster data acquisition^23^ but are not yet routinely available on all clinical MRI scanners.

Routine spectroscopic techniques cannot separate some important lower‐concentration metabolites, such as 2HG, GABA, GSH, and Glu from overlapping high‐concentration metabolites. In contrast, research applications, such as Mescher–Garwood (MEGA)‐point resolved spectroscopy (PRESS) and MEGA‐localized adiabatic spin‐echo refocusing (LASER) sequences, were implemented for GABA and 2HG editing to determine the genotype of a brain tumor.^20^ Also, there are some limitations in terms of 2HG acquisition. First, the absolute cutoff value of 2HG for predicting the IDH mutation is still debatable.^24^ Moreover, Glu and Gln peaks overlap with the peaks of 2HG in the spectral region between 2.1 and 2.4 ppm, making it more difficult to differentiate these metabolites using traditional approaches.^14^ A possible solution to this problem is using two‐dimensional MRS to differentially detect these overlapping peaks.^25^ In addition, 13C and 31P MRS/I require additional hardware, which has limited their clinical usage.

Molecular genetics has been more widely included in the classification of gliomas,^16^ and understanding the abnormal metabolism underlying genetic mutations using 1H‐MRS/I will become more important. Combined with diffusion MRI, DSC, and DCE methods, MRS can provide more information in >95% of cases before surgical excision and histopathological definition.^26^

## Summary

In vivo MRS provides noninvasive detection and quantification of tissue metabolites. It holds promise as a clinical tool through some metabolites (eg 2HG, Glx) that are already linked to positive and negative genetic prognostic factors included in the 2021 WHO classification. Relatively long data acquisition times and low SNR, however, hamper the feasibility of MRS/I in clinical settings. The wider availability of higher field strength scanners, phased array coils, accelerated techniques, and recent efforts toward consensus recommendations for MRS data acquisition and processing may help address these issues.

### Chemical Exchange Saturation Transfer

#### OVERVIEW

CEST imaging enables the acquisition of information from proteins, peptides, and small molecules, which are not detectable with conventional MRI due to their low concentration in tissue. Specifically, CEST selectively saturates the magnetization of solute molecules with exchangeable protons that resonate at a frequency different from water.^27^ This saturation results in a decrease in water magnetization, creating a new contrast associated with the solute pool.^28^ By exploiting the chemical exchange of exchangeable protons, CEST obtains indirect high‐resolution images from the solute pool.^29^ In a typical CEST sequence, a saturation period is followed by data acquisition,^28^ and the whole module is repeated while varying the saturation frequencies. Results are usually shown using a Z‐spectrum, which presents the measured normalized water intensity as a function of saturation frequency.^28^

Amide proton transfer (APT)‐CEST imaging is the most studied CEST technique and refers to effects observed around 3.5 ppm downfield from water.^27^ APT‐CEST is attributed to the slow‐exchanging amides in proteins and correlates strongly with pH.^27^ The nuclear Overhauser effect (NOE) is another CEST effect that arises from mobile macromolecules, observed at around −3.5 ppm.^29^

Amine protons at 2 and 3 ppm from water that exchange at intermediate and fast rates, respectively, are found in important molecules, such as creatine, glutamate, and proteins. The detection of these exchanging pools has potential practical applications in the brain (tumors and associated epilepsy), muscle, and heart, motivating the development of appropriate CEST methods.^30^

Glucose CEST (glucoCEST) relies on the injection of exogenous d‐glucose to study tissue perfusion parameters, such as blood volume, blood–brain barrier (BBB) permeability, as well as tumor malignancy, without the need for a GBCA injection. This method provides more reliable results at 7 T than at 3 T.^31^

Isolating CEST contrast in vivo while controlling for multiple confounding effects requires advanced postprocessing. A range of techniques is available, resulting in several potential metrics with which to describe the CEST effect. Asymmetry analysis (MTR_asym_) is an inherently simple approach, and its efficiency and ease of use have made this method popular in patient studies. However, different methods have been developed in response to the challenges encountered with MTR_asym_. These challenges include a macromolecular contribution due to the asymmetry of magnetization transfer effects and the contribution of NOE effects. Although a detailed description of these methods is beyond the scope of this article, it is important to mention the most promising ones: water saturation shift referencing (WASSR); the three‐offset method (APT*); MTR_REX;_ the apparent relaxation due to exchange (AREX); the apparent APT ratio (APTR*); and Z‐spectrum modulation as a combination of direct water saturation and solute pools of interest.^29^

#### CLINICAL APPLICATION

**FIGURE 3:**
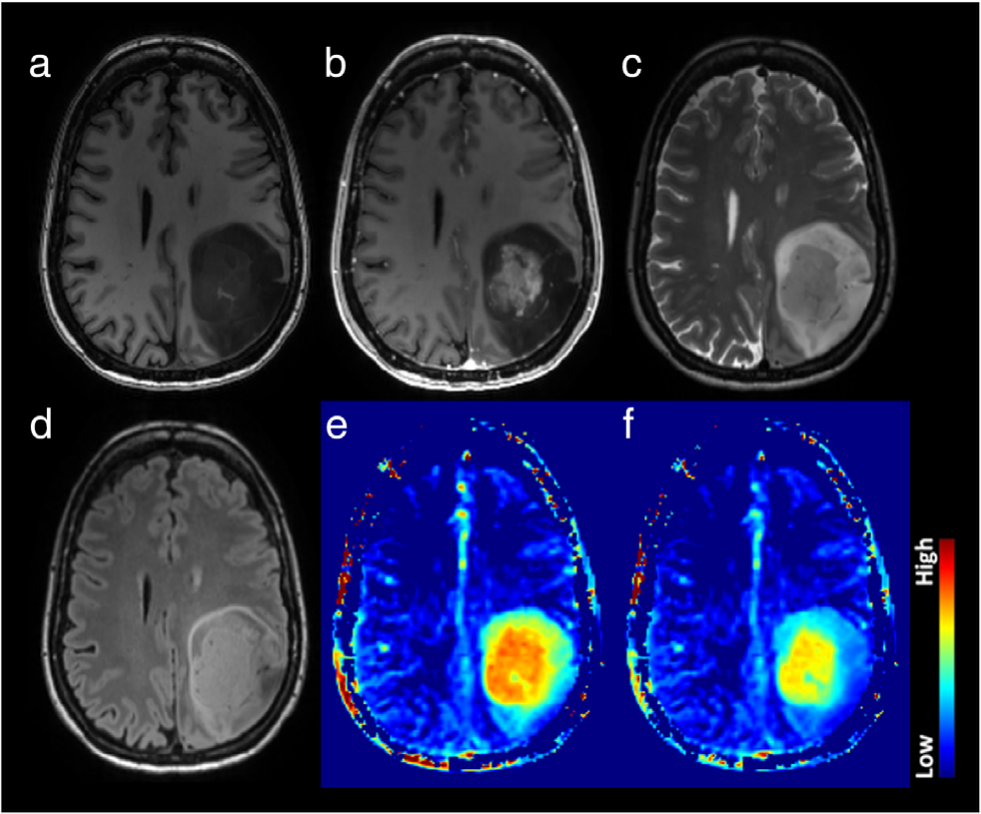
Example of an astrocytoma, IDH‐mutant, 1p/19q retained, CNS WHO grade 4. The structural images (a: T1w, b: T1w post‐Gd, c: T2w, d: FLAIR) demonstrate a heterogeneous lesion with a rather solid central and well‐enhancing part and a peripheral compartment demonstrating some T2/FLAIR mismatch without overt enhancement. The APT‐weighted maps (e: standard APT CEST, f: fluid‐suppressed APT CEST;Source: Casagranda S et al. ISMRM 29th An Meet 2021) show significantly elevated signal in the enhancing tumor, suggesting clearly high‐grade features. Notably, the rim zone of the lesion shows variable degrees of APTw signal elevation in the fluid‐suppressed images, thus suggesting that this compartment features mixed solid and cystic parts. Interestingly, the anterior rim zone, along with a halo surrounding the enhancing area, demonstrates a mildly elevated APTw signal that indicates likely high‐grade metabolic tumor characteristics. The data were acquired on a Siemens 3 T Prisma scanner. APTw protocol included DC = 91%, B1rms = 2uT, Tsat = 2 s, and WASAB1 for B0 correction. WASAB1 and APTw data were processed in Olea Sphere 3.0 software (Olea Medical, La Ciotat, France).

Because of its ability to reflect molecular changes, APT‐CEST is used to study tumor microenvironment and metabolism in vivo,^29^ as demonstrated in Fig. 3.

Cancer cells often exhibit structural, physiologic, and molecular changes and have an altered metabolic profile compared to healthy cells. Especially in high‐grade gliomas, the level of peptides and mobile proteins is substantially increased compared to surrounding tissue.^32^ An elevated protein content entails increased chemical exchange between the solute and bulk water. A good correlation has been demonstrated between endogenous protein profiles and APT‐weighted signals in gliomas.^33^ Studies that have assessed APT‐CEST have shown a sensitivity to differentiate tumor grades, with increased contrast in higher grades, and the ability to detect tumor aggressiveness.^34^ However, different studies have also shown that suppressing NOE contrast, often decreased in glioma compared to healthy‐appearing brain tissue, allowed more reliable characterization of the enhancing lesions of glioblastomas and differentiation between glioma grades, considering the IDH mutations and MGMT methylation status.^35^ Investigating CEST contrast in relation to molecular and genetic markers is in line with the most recent 2021 WHO classification.^36^

The potential usefulness of APT‐CEST for presurgical applications relies, in particular, on early detection and, consequently, propagation of more targeted treatment strategies, especially in the group of patients who do not show typical contrast enhancement on conventional T1‐weighted imaging, although they harbor HGGs.^37^ Recent work by Warnert et al aimed to use APT contrast to image nonenhancing gliomas and to more accurately distinguish tumorous from healthy tissue, based on tumor heterogeneity.^38^ Heterogeneous APT‐CEST contrast was detected within these tumors, with a greater effect size of APT‐CEST.^38^ Understanding the cause for the intratumoral contrast differences could include retrieving biopsies from APT‐hyperintense lesions to correlate with histopathological observations and improve overall diagnosis.^39^

Given the popularity and large body of work performed around this technique, recently published work has attempted to homogenize the application of APT‐CEST in available clinical systems.^40^

##### GlucoCEST

Since tumor cells utilize a glycolytic metabolic pathway, there will be an increase in glucose consumption. As such, glucoCEST imaging has been suggested to depict the saturation exchange between glucose‐hydroxyl protons and water between 1.2 and 3 ppm.^31^ Recent studies in glioma patients showed that the glucoCEST signal from dynamic glucose injection may reflect local blood flow, vascular permeability, and volume of the extracellular space, somewhat similar to what DCE T1‐weighted MR does although the correlation between DCE and dynamic glucoCEST cannot be fully understood at the moment.^41^

##### Amine CEST

Together with the glycolytic metabolism, the hypoxic microenvironment that is considered one of the major driving forces of tumorigenesis leads to intra‐ and extracellular acidosis in solid tumors, and these intracellular pH changes (pHi) may be evaluated using Amine‐CEST.^42^ In addition, it has also been shown that increased levels of amine protons can be detected in regions of an active tumor where mobile Gln and other neutral amino acids are a major source of fuel for malignant tumors, and transport systems are often amplified to increase Gln consumption.^43^

Specifically, the amine CEST contrast at 2 ppm has been shown to correlate with Cr distribution in brain tumors, which is an essential metabolite in the process of converting adenosine diphosphate (ADP) to adenosine triphosphate (ATP).^44^ A decrease in Cr CEST contrast was correlated with increased aggressiveness, and significant differences between the tumor and healthy brain regions have been observed, which most probably reflects the abnormal metabolism of gliomas in different malignancy states.^45^

It has also been suggested that the amine and amide concentration‐independent detection (AACID) signal from the ratio of the CEST effects generated by amide and amine protons from endogenous tissue proteins may be used to evaluate intracellular pH changes (pHi) in stroke.^42^

Moreover, the amines of Glu resonating at around 3 ppm have been shown to also play a role in CEST contrasts of gliomas. Neal et al have shown that an increase in Glu concentration in the peritumoral area of diffuse gliomas is a result of altered Glu homeostasis.^46^ Altered Glu concentrations were associated with higher glioma aggressiveness, described by the enhancement on contrast‐enhanced scans.^46^

#### VALIDATION

CEST, including APT, has not yet been widely implemented in clinical settings for glioma imaging. However, in a recent consensus publication, updated implementation guidelines have been defined. There has also been an effort from the industry to develop a clinical sequence, which has resulted in a commercially available APT‐CEST product for clinical use. Yet, cross‐vendor reproducibility has not been widely investigated. Most studies have, so far, focused on technical validation and, to some extent, have included clinical validation; however, a sizable multi‐site comparison is still missing. Another challenge includes the lack of standardized diagnostic cut‐off criteria, which would be essential for wide clinical use. Last, implementation, including data analysis and postprocessing, would require special training and expertise. Once these translational challenges are tackled, CEST could be an interesting technique to adopt in glioma imaging.

#### SUMMARY

In conclusion, CEST has shown potential as a novel technique that can provide unique endogenous contrast. APT‐CEST yields the most promising results, evidenced by its popularity and high research output. Other CEST‐based contrasts that derive from amine and glucose still need to demonstrate their value in larger cohorts. Overall, CEST is still in need of multisite, multivendor clinical validation before it can be adopted for widespread glioma imaging in clinical practice.

### Susceptibility‐Weighted Imaging

#### OVERVIEW

**FIGURE 4:**
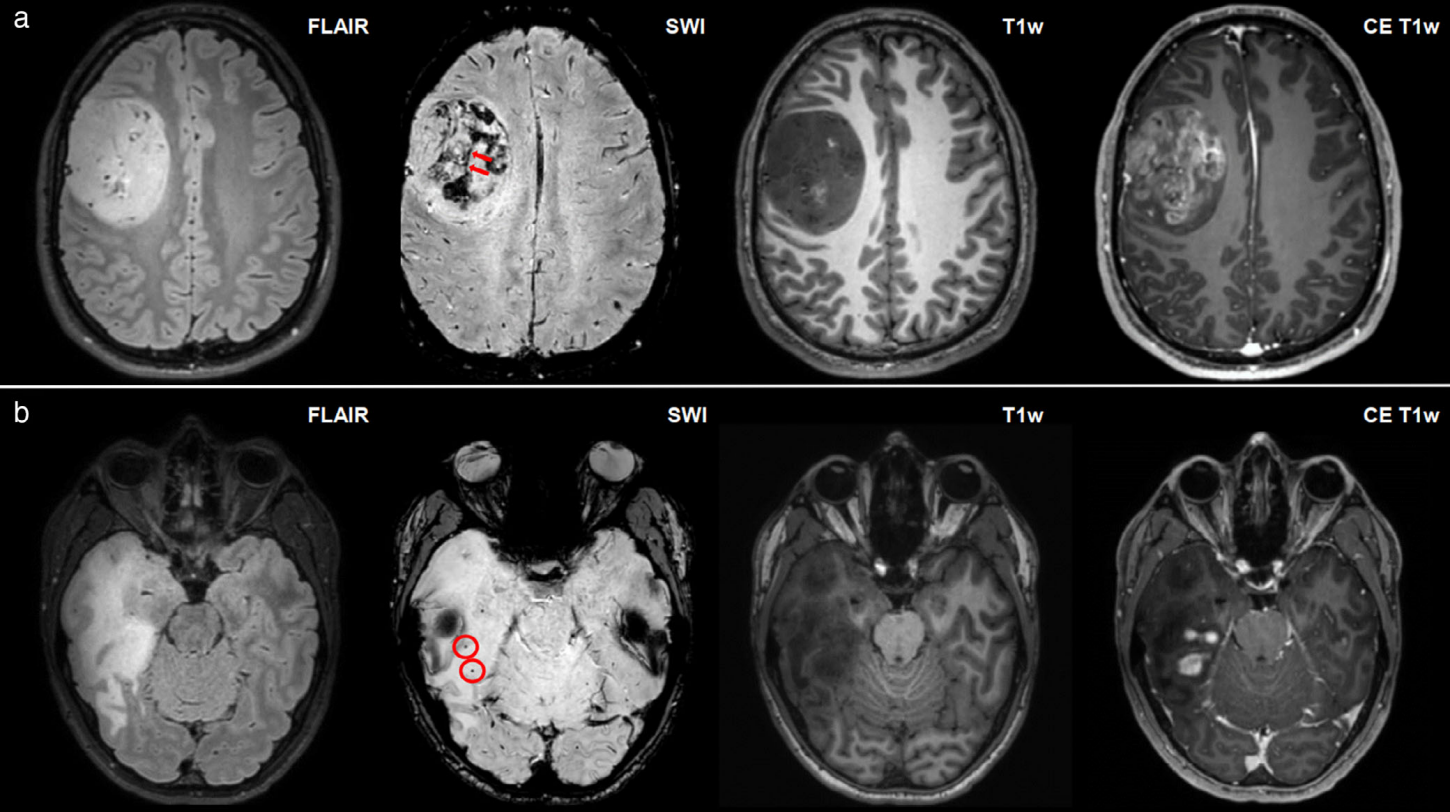
Panel (a) depicts a right frontal glioblastoma in a 40‐year‐old male and panel (B) depicts a right temporal anaplastic astrocytoma in a 32‐year‐old female, with high signal on fluid‐attenuated inversion recovery (FLAIR), low signal on T1‐weighted imaging, and partial contrast enhancement according to contrast‐enhanced T1‐weighted imaging. Intratumoral susceptibility signal (ITSS) abnormalities can be found according to susceptibility‐weighted imaging (SWI) in both patient cases (a: multiple dot‐like and fine linear ITSS abnormalities corresponding to ITSS grade 3, red arrowheads point at a prominent linear ITSS within the lesion; b: only few dot‐like ITSS abnormalities corresponding to ITSS grade 1, red circles enclose two exemplary dot‐like signal drops within the lesion).

SWI is a high‐resolution qualitative MRI technique that assesses susceptibility and T2* differences between tissues.^47^ Next to a magnitude image, an SWI‐filtered phase image is created, which filters out artifacts and visualizes the direction of phase shift caused by diamagnetic (eg calcium) and paramagnetic (eg deoxygenated hemoglobin) substances. Calcifications, (micro)hemorrhages, and neovascularization are the main sources of signals in glioma.^48^ Assessment is usually visual. The intralesional/−tumoral susceptibility signal (ILSS or ITSS) is a proposed visual grading index derived from standard SWI^49^ (Fig. 4). However, SWI can be adapted to generate quantifiable measurements (QSM, quantitative susceptibility mapping). SWI can be obtained at 1.5 T as well, although this might prolong the acquisition time.

#### CLINICAL APPLICATION

The utility of SWI to differentiate glioma types based on a correlation with histology was first documented in 2007^50^ and was confirmed in subsequent studies,^51^ but recent updates according to the 2021 WHO classification are scarce. Generally, the lower the glioma grade, the fewer punctiform or linear vessel signals are found, that is, the lower the ITSS.^52^ Wang et al reported significant correlations of ITSS within astrocytomas, with relative cerebral blood volume (rCBV) max (*r* = 0.92) and with tumor grades (*r* = 0.92), suggesting a combination of SWI and dynamic susceptibility contrast (DSC) could improve the diagnostic accuracy of astrocytoma grading.^53^ Quantitative SWI approaches, for example, using parameter ITSS‐vasculature volume (IVV), may better differentiate tumor vessels from microhemorrhage and improve tumor grading.^54^ In a 2020 study, IVV provided the highest AUC for the discrimination of grade II vs. III (0.93), grade III vs. IV (0.98), and grade II vs. IV (0.94) compared to other semi‐quantitative scoring approaches. IVV also provided the highest sensitivity and specificity for differentiating grade II vs. III (87.44, 98.41), grade III vs. IV (97.15, 94.12), and grade II vs. IV (98.72, 92.31). The multicenter study by Saini et al found that a combination of rCBV and SWI‐derived ITSS improved the diagnostic accuracy for discrimination of grade II/III from grade IV gliomas.^55^ The 1p/19p co‐deletion status of IDH‐mutant LGG to identify oligodendrogliomas could be predicted using a combination of parameters, including SWI at an AUC of 0.88 in another large study.^56^

SWI can be applied after GBCA administration, and this may even have additional diagnostic value, as accumulating contrast agents can enhance the T2* effect, which is reflected in the SWI image.^57^ One study proposed that the different tumor margins seen after contrast‐enhanced SWI represent the tumor invasion zone outside the core tumor area,^58^ while another study showed its capacity to differentiate HGG from metastasis.^59^

**FIGURE 5:**
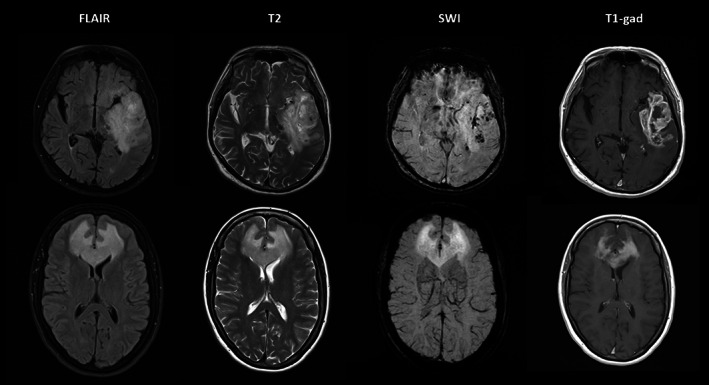
Top row: 74‐year‐old man presenting with aphasia was found to have a high‐grade left temporal glioma on MRI (1.5 T). Note the intratumoral susceptibility signals (ITSS) on SWI indicative of microhemorrhage and vessel proliferation. Bottom row: 57‐year‐old woman presenting with behavioral changes due to a lymphoma. SWI does not show any ITSS despite marked homogeneous enhancement.^62^

Other successful SWI applications to differentiate glioma from other lesions exist: Lai et al. differentiated abscess from necrotic gliomas using SWI and ADC either separated or combined,^60^ and concluded that ITSS combined with ADC showed a 100% diagnostic accuracy in differentiating abscesses from glioblastoma, SWI and ADC being complementary. Peters et al reported that, by using SWI, radiologists were able to differentiate between glioblastoma and primary CNS lymphoma in 82.2% of the cases, while, without SWI, the diagnosis was correct in only 75.5% of the cases^61^ (Fig. 5). Recently, Ozturk et al reported that a combined analysis of SWI and DWI could differentiate atypical glioblastoma from primary CNS lymphoma, including molecular criteria using scores relative to the contralateral hemisphere (rSWI): glioblastomas without the IDH1 mutation demonstrated a significantly lower rSWI value compared to glioblastomas with an IDH1 mutation and PCNSL.^63^ The incorporation of ADC and SWI parameters distinguished glioblastoma with IDH1 mutations with a sensitivity and specificity of 94.3% and 100%, respectively.^63^

#### VALIDATION

SWI is a product sequence for all major MRI vendors. The necessity to know the handedness of phase images by vendors remains an obstacle in the interpretation of images. SWI acquisitions have been verified in multivendor and multicenter trials for 1.5–7 T and compared between precontrast and postcontrast acquisition. It is a widely used and clinically accepted sequence.

#### SUMMARY

SWI is a clinically available technique with relatively low‐threshold prerequisites for interpretation and ample documentation of added value for glioma differentiation.^51^ Despite a lack of large and controlled studies that use the most recent diagnostic criteria for gliomas, SWI is readily available and may therefore be considered underused in clinical practice.

### MRI‐PET

#### OVERVIEW

MRI‐PET combines PET and MRI into a single system to visualize both structure and function. PET uses radioactive tracers to reflect the (patho)physiological processes at the molecular level. Although PET has demonstrated high accuracy in measuring metabolic activities with certain tracers, it lacks detailed anatomical information in the scan. MRI offers the advantage of producing high‐resolution anatomical scans with detailed soft tissue contrast.^64^

While separate or sequential MRI‐PET systems exist, most new systems have an integrated configuration in which the PET detectors are inside the MRI gradient coils, allowing simultaneous MRI‐PET data acquisition. For PET attenuation correction, an MRI scan is used instead of CT. The MRI scan is segmented to soft tissues and bones and used to create tissue density maps for attenuation.^65^

**FIGURE 6:**
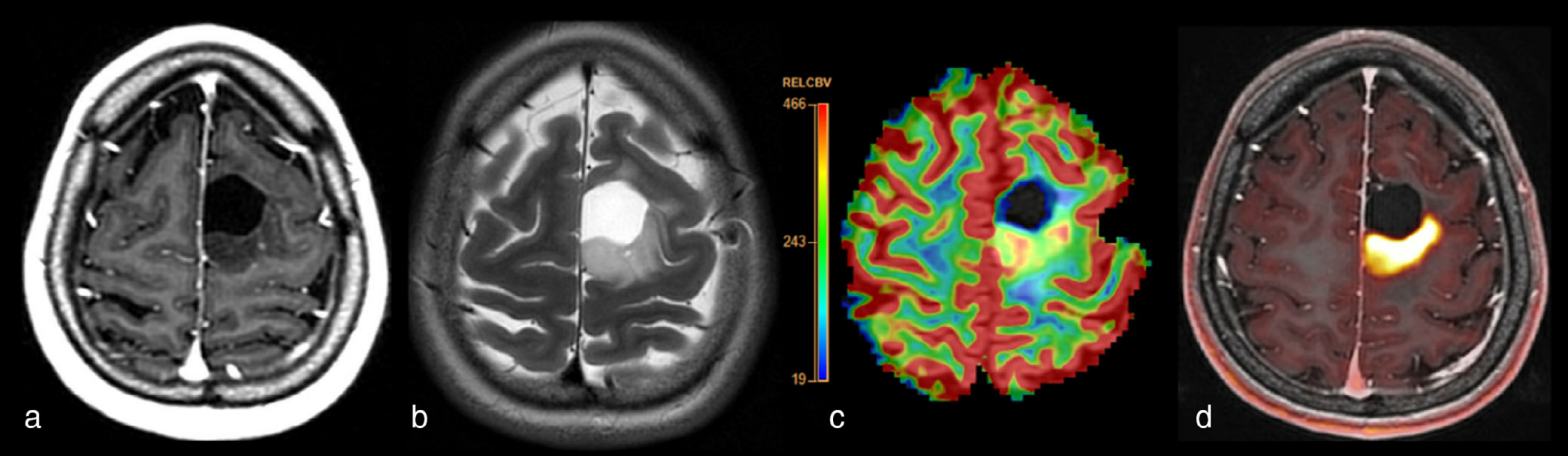
F^18^‐FET‐PET‐MRI of a 28‐year‐old female patient with resection of oligodendroglioma and a growing nonenhancing lesion posterior from the resection cavity with concordant high perfusion and high FET‐uptake consistent with (histopathologically confirmed) low‐grade recurrence. (a) Postcontrast T1w, (b) T2w, (c) rCBV map and (d) FET‐PET overlaid on postcontrast T1w.

The high physiological uptake of ^18^F‐fluorodeoxyglucose (FDG), a commonly used radiotracer, in normal gray matter limits its utility for gliomas. There is growing evidence for the utility of amino acid tracers that target l‐amino acid transporter systems 1 and 2.^66^, ^67^, ^68^ The most frequently used tracers for glioma imaging are ^11^C‐methyl‐methionine (MET) and O_2_‐^18^F‐fluoroethyl‐l‐tyrosine (FET), as shown in Fig. 6. These radiolabeled amino‐acid analogs have similar cellular mechanisms and are able to detect increased amino‐acid transport and protein synthesis as signs of high cellular proliferation in tumors.^69^ Recently, practical guidelines on the acquisition, reconstruction, quantification, and cut‐off thresholds for biological tumor volume were published to facilitate clinical translation.^68^

#### CLINICAL APPLICATION

The role of PET in the diagnosis of neoplasms is reflected by the significantly higher uptake of neoplastic lesions compared to non‐neoplastic lesions.^70^ According to the guidelines by Law et al, a negative FET/MET/FDOPA‐PET scan (with uptake in the background uptake range or slightly above) excludes grade III/IV, lymphoma, or metastases with a high probability, while increased uptake has a high positive predictive value for a neoplastic process.^68^ However, all PET tracers also may show increased uptake in inflammatory lesions, in the context of an epileptic seizure, or in hemorrhagic or ischemic lesions, which are the most common pitfalls in terms of neoplasm diagnosis.^68^ PET imaging has a further impact on the differentiation of gliomas from other tumor entities. In terms of glioma grading, a meta‐analysis by Katsanos et al, including a total of 994 participants, showed significantly higher pooled sensitivities for differentiating HGG from LGG for both MET PET and FET PET, compared to FDG PET, while FDG PET was superior in terms of specificity.^71^

PET may also provide prognostic information. Suchorska et al assessed 300 patients with WHO grade II‐IV gliomas, grouped according to IDH1/2 mutation and 1p/19q codeletion, and showed that dynamic FET PET may provide further prognostic information in IDH1/IDH2 mutant diffuse gliomas, independent of WHO grading.^72^ Moreover, Kunz et al evaluated 98 patients with nonenhancing glioma, classified according to 2016 WHO classification, and showed that dynamic FET PET can provide prognostic information independent of WHO grade and IDH mutational status.^73^

Specifically, there may be an added value in the combination of amino‐acid PET and conventional MRI to outline glioma infiltration, which has been demonstrated to extend beyond what can be outlined on conventional MR images. This, in turn, affects biopsy planning as the metabolically active tumor appears larger on PET than the area of MRI contrast enhancement in pre‐surgery imaging.^74^, ^75^ FET can be combined also with advanced MRI, such as DSC, and such a combination can further improve the distinction between IDH‐mutated astrocytomas and IDH‐wildtype glioblastomas.^76^

While PET imaging can provide additional metabolic and physiological information to conventional MRI, advanced MRI sequences, for example, APT‐CEST, MRS, DWI, and DSC/DCE, can provide similar information to that of PET on tumor biology. However, head‐to‐head comparisons of advanced MRI and PET in the context of neuro‐oncology are sparse, and future research is warranted to compare their diagnostic potential.

#### VALIDATION

MRI‐PET may serve as a clinical decision tool for patient management in glioma imaging, as it can provide synchronous structural and metabolic information. Despite the promising advances, a number of technical and procedural challenges exist before clinical application. For example, evidence has suggested that the genotoxic potential of ionizing radiation increases in the presence of a static magnetic field, as in MRI‐PET.^77^ Due to the simultaneous acquisition of PET and MRI data, image registration and motion correction have been significantly improved using the anatomical information of MRI.^78^ However, the associated benefits in patient management remain to be evaluated.

#### SUMMARY

MRI‐PET has technically come of age and scanners are now more frequently available in larger centers. Hybrid scanners obviate, in theory, an additional patient visit for the PET examination that warrants a widespread clinical use. However, the availability of the essential amino‐acid tracers is limited due to their short half‐life, and their integration into clinical routine is therefore still limited. The added value of amino‐acid PET in comparison to advanced MRI still needs to be established.

### MR Elastography

#### OVERVIEW

Many histopathological processes in tumors can cause changes in the viscoelastic properties of tissue, such as cell proliferation, angiogenesis, fibrosis and necrosis, and cyst formation. Against this background, MRE is a technique by which to noninvasively measure the biomechanical properties of tissue. In MRE of the brain, a vibrational device is placed on the patient's head, causing a shear wave to pass through the brain tissue. This motion is imaged using a modified phase‐contrast MR sequence with motion‐encoding gradients. Viscoelastic maps are then calculated using an inversion algorithm. The measured value is the shear modulus G*, which describes the elastic and viscous properties of the tissue. The magnitude of the shear modulus |G*| is commonly used as a measure of tissue stiffness. The shear phase angle, *φ*, describes the viscous tissue properties, with a higher phase angle indicating a more complex tissue structure.^79^

#### CLINICAL APPLICATION

**FIGURE 7:**
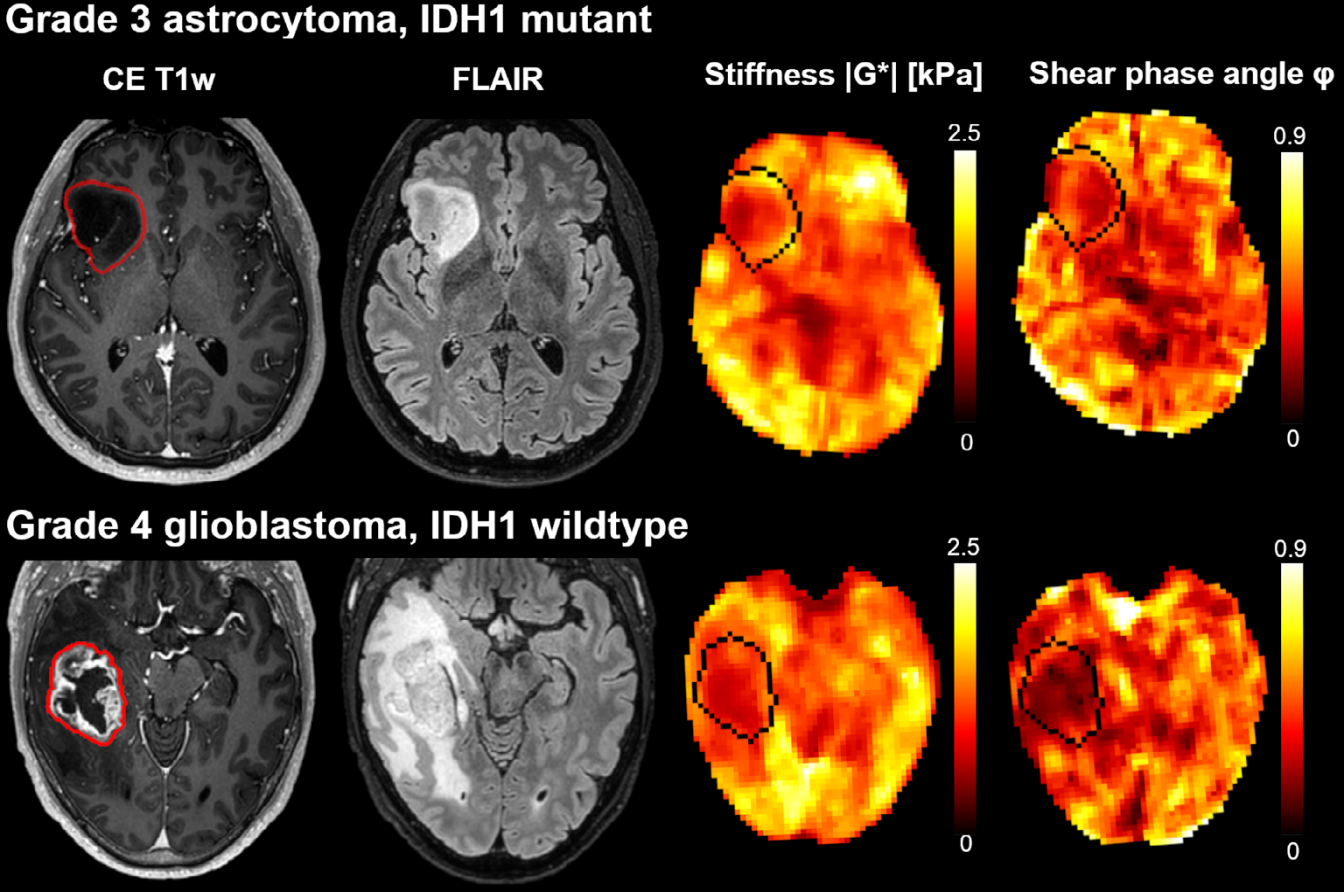
Stiffness heterogeneity of gliomas. Contrast‐enhanced T1‐weighted images, FLAIR images, |G*| (stiffness), and φ maps (phase angle, related to viscosity) for two patients with gliomas. The images in the upper row are derived from a 40‐year‐old man with an IDH1‐mutated grade 3 astrocytoma, and the images in the lower row are derived from a 55‐year‐old man with an IDH1‐wild‐type grade 4 glioblastoma.

Studies of MRE used in patients with glioma have found that gliomas are predominantly softer than normal‐appearing white matter (NAWM).^80^, ^81^, ^82^, ^83^, ^84^, ^85^ In a review of MRE in patients with brain tumors, the softening of gliomas compared to NAWM was calculated across published studies: the mean stiffness reduction was 17% in glioblastomas (*n* = 36), 14% in WHO grade 3 astrocytomas (*n* = 5), and 34% in low‐grade gliomas (*n* = 5).^86^ However, there are reports of WHO grade 3 and 4 gliomas with stiffness higher than NAWM, possibly illustrating the between‐tumor heterogeneity of high‐grade gliomas.^82^ The average decrease in phase angles compared to NAWM was 30% in glioblastomas, 4% in grade 3 astrocytomas, and 1% in low‐grade gliomas^86^ (Fig. 7).

In contrast, for cancer outside the brain, there is an apparent consensus that solid tumors are associated with tissue stiffening.^87^ However, the brain itself is also much softer than other body tissues.^88^ Softening in tumors may be due to a reduction in the structure of the cross‐linking network.^81^ Rapid and chaotic tumor cell growth reduces structural anisotropy in the brain, leading to lower viscoelastic properties of brain tumors.^89^ Furthermore, necrosis leads to tissue liquefaction, reflected by reduced viscoelasticity in necrotic tumor regions.^85^, ^90^ The abnormally low phase angle values measured in glioblastomas may suggest that the fluid properties of the tumor are part of the infiltrative tumor growth.^84^ Svenson et al have shown that abnormal tissue properties were present in regions that appeared normal of conventional MRI.^85^

Three studies have investigated the use of MRE in the characterization of glioma, and all found glioblastomas to be softer than gliomas of lower grades.^81^, ^82^, ^83^ In a piliot study studying various intracranial tumors, primary brain tumors and cerebral metastases were not distinguishable in terms of |G*| and φ.^81^ No significant stiffness differences have been found between lower‐grade gliomas. Phase angles were reduced in all gliomas, with mean phase angle values decreasing with higher tumor grades.^82^ Moreover, IDH1‐mutated gliomas are significantly stiffer than IDH1 wild‐type gliomas, regardless of tumor grade.^83^

Most studies of MRE in glioma present mean tumor values. This may, in part, be explained by the relatively low resolution of MRE imaging (currently limited to 2–3 mm in‐plane resolution) and further spatial averaging in the calculation of viscoelastic parameters.^91^ Glioblastoma stiffness maps are typically heterogeneous, with tumors being composed of stiff and soft compartments.^80^, ^85^ Stiffness and viscosity are lower in necrotic areas than in the contrast‐enhancing parts of the tumor.^85^

#### VALIDATION

Brain MRE is not currently used in the clinical routine for glioma imaging. Based on the overlapping distribution of reported stiffness measurements across different brain tumors, MRE may not be suitable for the discrimination of different brain tumor types in a clinical setting.^86^ However, the phase angle might serve as an alternative and more sensitive measure of malignancy; thus, future studies should report on both stiffness and viscosity parameters. Furthermore, the technique has been used and validated in clinical studies of Alzheimer's disease, multiple sclerosis, Parkinson's disease, and normal pressure hydrocephalus.^92^ In general, no significant risks are associated with the MRE technique, and patients with brain tumors tolerate the mechanical vibrations of 30–60 Hz well.^85^ The scan durations typically range between 5 and 10 minutes.

While repeatability was good within each technique, the reproducibility of tissue stiffness estimates between sites remains challenging.^93^ Tissue stiffness estimates may vary with MRE hardware, vibration frequency, acquisition methods, and processing pipelines.^92^ It is recommended that tumor stiffness and viscosity relative to each patient's NAWM be measured.^79^ A continued focus on hardware, acquisition, sequences, and reconstruction methods will bring this technique closer to clinical viability.

#### SUMMARY

The tissue stiffness and viscosity of a glioma tumor and its microenvironment is altered compared to healthy tissue. MRE is uniquely able to measure these changes in biomechanical properties in vivo and therefore holds promise as a relevant clinical tool. However, based on the handful of available studies using MRE in glioma, the technique is still in development, and multicenter studies are needed to support its use for discriminating between different brain tumor types and stages.

### Radiomics

#### OVERVIEW

**FIGURE 8:**
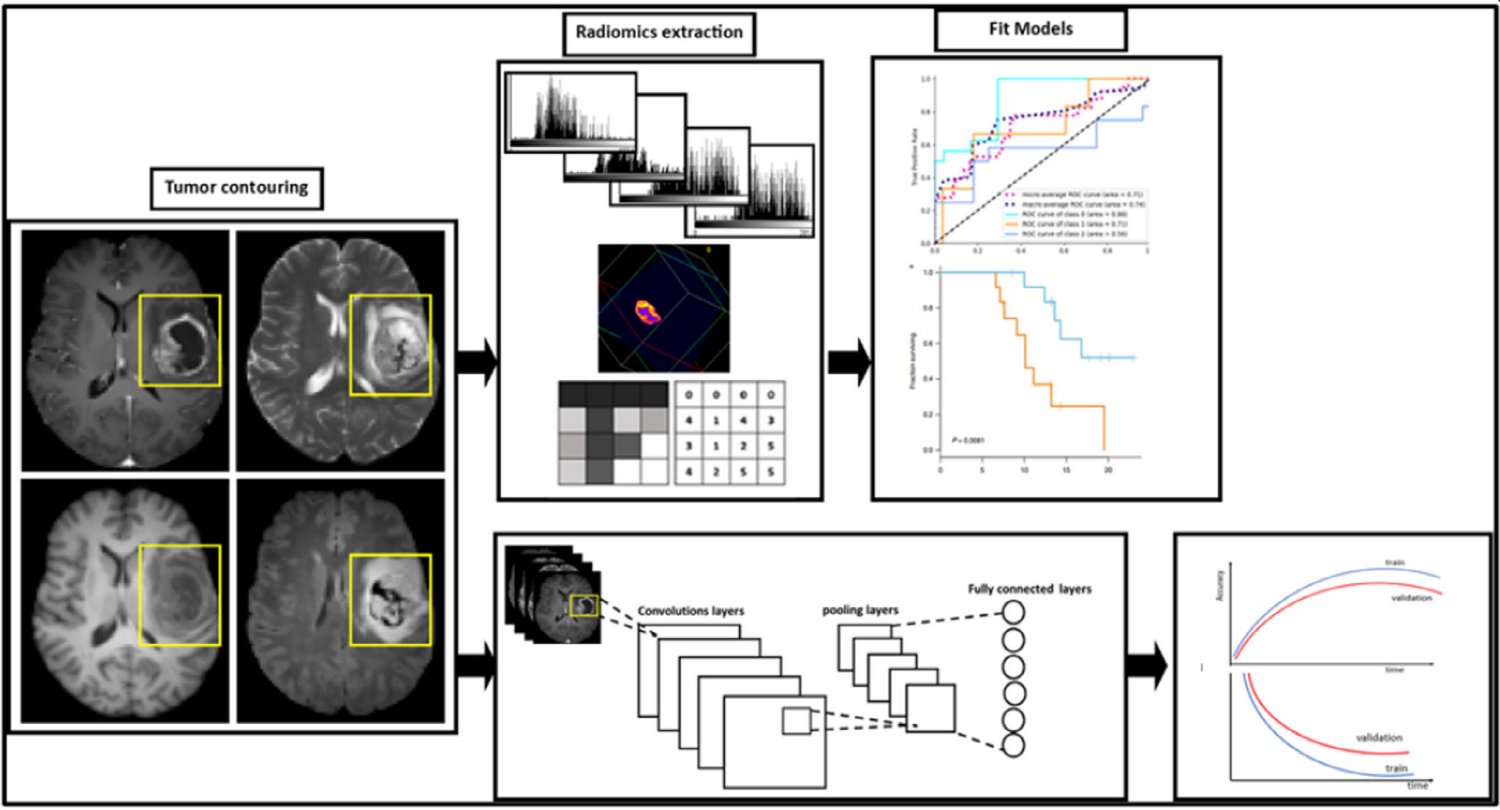
Schematic representation of classic machine learning (top) and deep learning (down) for imaging data from gliomas.

Artificial intelligence (AI)‐assisted radiomics is an approach that extracts quantitative imaging features to be used in machine‐learning (ML) prediction models.^94^, ^95^ This approach allows pattern recognition on a large number of quantitative features and is less subjective and faster compared to visual evaluation. Multiple radiomics approaches have been proposed for the non‐invasive and accurate grading of gliomas using features extracted from multiparametric MRI, such as histogram and texture features with a support vector machine (SVM) algorithm.^96^ More recently, a hybrid radiomics approach, using a random forest classifier showed improved grading accuracy compared to the results of similar studies.^97^ The advantage of these classical ML algorithms is the ability to handle a sample‐size classification problem ably. However, some meaningful features may remain unnoticed, and using deep learning‐based models (DL) might offer more flexibility in learning discriminative high‐level features when larger training datasets are available (Fig. 8). While AI‐driven methods can also be used for MRI acquisition, postprocessing, and analysis in general, this lies beyond the scope of this review and is covered by elsewhere.^98^, ^99^

#### CLINICAL APPLICATION

Most of the radiomics studies focus on working with the conventional structural and diffusion MRI data as these are acquired in clinical routine, allowing easy translation and securing large datasets needed for training and validation. Molecular biomarkers have gained further importance in the latest WHO 2021 guidelines for glioma classification. IDH‐mutant gliomas have consistently demonstrated less pronounced imaging features, including higher ADC and lower rCBV, than IDH wild‐type gliomas.^100^

Lasocki et al examined multiple conventional MRI features for the prediction of the three key molecular subtypes, IDH‐mutant and 1p/19q co‐deleted, IDH‐mutant and 1p/19q intact, and IDH‐wildtype in grade 2 and 3 gliomas using multivariate logistic regression analysis, and reported T2‐FLAIR mismatch as the most predictive feature across the three genotypes.^101^ Additionally, contrast enhancement, hemorrhage, and necrosis were observed to be correlated with IDH wild‐type status, while calcification was found to be correlated with IDH mutant and 1p/19q co‐deleted status.

Other imaging studies have identified further molecular markers important in predicting glioma tumor biology. Pease et al developed a pre‐operative MRI‐based radiomics model to find EGFR amplification, MGMT methylation, and molecular subgroups in glioblastoma patients.^102^ Their study achieved AUC values greater than 0.83, 0.85, and 0.92 for predicting EGFR, MGMT, and molecular subtypes, respectively.

In addition, an image fusion model that included radiomics signatures from T1‐weighted contrast‐enhanced imaging and ADC achieved AUCs of 0.884 and 0.669, respectively, to predict IDH and TERT status.^103^ Ahn et al conducted a study in which gliomas with IDH and tumor protein 53 (TP53) mutations and alpha‐thalassemia/mental retardation X‐linked (ATRX) loss were found to be clustered according to their shared imaging features, including a poor definition of enhancing margin, high ADC values, and a higher proportion of T2 hyperintense lesions.^104^ In a multicenter study, Ali et al set to overcome the scanner‐dependent domain mismatches using an unpaired‐Cycle Generative Adversarial Network (CycleGAN). Their results showed that unpaired CycleGAN mitigated the domain differences while maintaining the subtitle‐molecular information, with a noticeable increase in the performance compared to when the dataset was not mapped (74.81%, improved by 7.78% on 1p/19q codeletion status and 81.19%, improved by 8.81% on IDH mutation status).^105^

Van der Voort et al developed an algorithm with data from 1,508 patients which simultaneously segments, grades, and genotypes of glioma in terms of IDH mutation and 1p/19q codeletion. The model reached accuracies of 80%–90% in an entirely independent dataset of 240 patients, which were similar to those reached in the development set, evidencing the robustness of the algorithm against scanner, site, and protocol variations.^106^

As the contrast‐agent injection is part of clinical protocols and DSC is highly validated, research protocols commonly include DSC. This makes DSC datasets fairly available in glioma datasets. IDH mutation status in gliomas has been found to be noninvasively predictable with rCBV on DSC‐MRI using ML approaches. A leave‐one‐out cross‐validated logistic regression model correctly predicted IDH mutation status in 88% of LGG patients.^107^ A generalized linear model classifier combining DSC and DWI reached an AUC of 0.795 in predicting IDH status in LGG^108^ and 0.88 and 0.76 in predicting receptor tyrosine kinase and tumor protein p53, respectively, in IDH wild‐type glioblastomas.^109^

In contrast, most methods of advanced MRI are commonly applied in single‐center smaller studies only, complicating the use and validation of their use in radiomics. As reviewed in the first part of this article, arterial spin labeling (ASL) is a viable noninvasive alternative for DSC.^110^ Similar performance of ASL and DSC in discriminating LGGs and HGGs was also shown when using radiomics analysis of these two sequences.^95^ Calabrese et al^94^ predicted the molecular biology of gliomas from conventional MRI data with ASL using a combination of radiomics and CNN features and achieved AUC‐ROCs of 0.97 for identifying ATRX loss, 0.96 for IDH1 mutation, 0.85 for TERT mutation, 0.80 for EGFR amplification, 0.79 for TP53 mutation, and 0.77 for MGMT promoter methylation. Using a combination of precontrast and postcontrast T1‐weighted images, T2‐weighted, multi‐b‐value diffusion‐weighted, and ASL images and textural features, Tian et al were able to achieve an AUC of 97% when discriminating HGGs and LGGs.^111^ Necrotic volume percentages of core (CNV), age, choline‐to‐creatine ratio, lactate, and a radiomics score were found to be significantly higher in TERT‐mutant than in TERT wild‐type high‐grade gliomas in a study using conventional MRI together with MRS.^112^ Overall, these studies are done in limited patient populations only (<200 subjects) without external validation. More work is likely needed to make the results generalize to data acquired at different scanners with different acquisition parameters.

#### VALIDATION

In general, a supervised machine‐learning model requires a curation process for data to train, validate, and test algorithms optimally. Single centers typically provide a limited dataset and information, which might bias the training, and, therefore, larger multicenter data studies should be conducted to allow the training of well‐generalizable models and properly assess this ability on heterogeneous data. Li and Huang^113^ and Eun Park et al^114^ emphasized the importance of an ongoing collaborative effort between research institutions, clinicians, and governing bodies, such as ISO and IEEE, to maximize the reproducibility and generalisability of AI models and radiomic features, and avoid over‐standardization in neuro‐oncological imaging and radiomics. Postprocessing and feature extraction will help to lower data‐induced incompatibility in radiomics models. A major effort in this direction was done by the Image Biomarker Standardization Initiative, which created a nomenclature and processing schemes for radiomics, and provided a set of 169 reference values for radiomics features. These reference values enable verification of radiomics software, which will increase the reproducibility of radiomics studies and facilitate clinical translation of radiomics.^115^

However, over‐standardization of methodologies by following strict guidelines could limit the potential of ground‐breaking discoveries unless the guidelines are constantly revised and updated to keep pace with technological developments. In addition, due to the unequal occurrence of glioma types, the classification models can have poor predictive performance for the types that are under‐represented in the training datasets. One way of overcoming such issues, that is, by working with highly diverse and very large datasets, is to use a federated learning approach. In the largest study to date for automated glioma segmentation, data from over 6300 patients from 71 institutions across the globe were used to achieve substantial improvement in terms of accuracy and robustness compared to previous approaches.^116^

Apart from conducting retrospective studies and generating concept models, the feature reproducibility and clinical utility of such models should be validated using prospective studies in a clinical setting. This would enable the building of more standardized protocols for real‐world implementations. The transparency of the AI models, otherwise known as explainable artificial intelligence (XAI), is immensely important as opposed to a black‐box approach when it comes to clinical operation.^117^

#### SUMMARY

Despite its potential, machine‐learning models are still facing issues with generalization to different centers' data and lack of evidence for clinical utility, which is the main obstacle to implementing and developing AI clinical applications. Large multicenter studies are needed to develop and validate.

## Discussion

In this review, a working group of the GliMR COST action summarizes the evidence for the clinical use of advanced MRI for preoperative glioma characterization. Posttreatment effects^5^, ^118^ and adverse treatment effects^119^ have been reviewed previously by GliMR members. A general discussion is included in the first part of this review [note: reference during layout].

Of the methods reviewed in this part, intriguingly, SWI is arguably the most commonly used method due to the simplicity of acquisition. Yet, it is not at the level of MRS in terms of technical and clinical evaluation. The combination of high accessibility and relatively unique outputs of SWI, therefore, speaks to what constitutes an advanced MRI technique that is clinically available and in use. In contrast, MRS or MRE also produce distinctive outputs but are technically challenging and hence currently offer lower levels of evidence for implementation into clinical routine.

While technically demanding, the unique information on tumor metabolic activity obtained with MRS and MRSI fits one of the most important changes in the new 2021 WHO classification; the mutation status of the IDH enzymes. The presence of IDH mutation has been found to be an inciting event in IDH‐mutant glioma tumorigenesis, with a strong effect on the oncogenic progression and clinical outcome.^120^ IDH mutation detection by MRS through the 2‐hydroxyglutarate oncometabolite has a high promise for a noninvasive glioma classification. To this end, clinical translation of MRS should be a priority with focused efforts to increase its level of clinical validation while reducing the current user dependency.

The development of hybrid MR‐PET machines offers the advantage of obtaining a PET measurement with good quality within the timeframe of MRI examination and thus without increasing the burden for the patient. However, owing to costs, logistics, and administrative reasons, hybrid machines are favored in larger centers and mainly for use in research. Moreover, the commonly used FDG tracer has a low specificity in brain tumors, and amino‐acid‐based tracers like FET and MET suffer from limited availability that prevents clinical use on a wider scale. CEST‐MRI is on a good path to provide a feasible and affordable alternative for imaging amino‐acid metabolism pending further technical development and clinical validation. Further research comparing PET to advanced MRI is necessary to establish the complementary roles of these two modalities for glioma imaging.

The largest potential for future development of advanced MRI lies in radiomics, although the current level of clinical validation is poor. A reason for this paradox is the need to curate larger datasets in order for the models to reach a sufficient level of maturity and hence validation. Moreover, while radiomics and AI play a major role in over half of the submissions in medical imaging journals like *JMRI*, a large portion of this research suffers from the domain gap between computer science and medicine and medical imaging, making the results less accessible to the medical community due to different writing styles and language. By investing in efforts aiming to connect the two domains, this untapped potential can be capitalized on.

In conclusion, the continued progress of advanced imaging techniques extends the possibilities of MRI to map the biological features of glioma (Table 2). By introducing these advanced techniques into MRI clinical protocols, they can provide diagnostic biomarkers with diverse predictive or prognostic values tailored to pertinent clinical questions. Combined with radiomics and artificial intelligence algorithms, advanced MRI may further enhance the clinical significance of imaging biomarkers toward more personalized and, hopefully, more effective therapies for glioma.

**TABLE 2:** Summary of Clinical Applications for the Prediction of Molecular Subtypes in Gliomas as Presented in This Review

Methodology	Parameters	Molecular marker	References
MRS	2HG	IDH mutation	13, 17
MRS	Multiple	IDH mutation	15
MRS	Cho, Glx	TERT mutation	15
CEST	APT	IDH mutation	35
CEST	APT	MGMT methylation	35
SWI	Multiple	1p/19q co‐deletion	56
SWI	rSWI	IDH mutation	63

The application of radiomics to this end is extensively discussed in the respective part.

## Supporting information


**Appendix S1:** Supplementary Information
