# Supplementary material for: Advanced MR Techniques for Preoperative Glioma Characterization: Part 2
Source: J Magn Reson Imaging. 2023 Mar 13;57(6):1676–95. doi: 10.1002/jmri.28663 (PMC10947037; doi:10.1002/jmri.28663)
Supplement: Supplementary file 1 — Appendix S1: Supplementary Information [file JMRI-57-1676-s001.docx]

# Supplementary materials

## References for the Level of Validation Table - MRS

Gasparovic, C., Bedrick, E. J., Mayer, A. R., Yeo, R. A., Chen, H., Damaraju, E., Calhoun, V. D., & Jung, R. E. (2011). Test-retest reliability and reproducibility of short-echo-time spectroscopic imaging of human brain at 3T. Magnetic Resonance in Medicine: Official Journal of the Society of Magnetic Resonance in Medicine / Society of Magnetic Resonance in Medicine, 66(2), 324–332.

Gu, M., Kim, D.-H., Mayer, D., Sullivan, E. V., Pfefferbaum, A., & Spielman, D. M. (2008). Reproducibility study of whole-brain 1H spectroscopic imaging with automated quantification. Magnetic Resonance in Medicine: Official Journal of the Society of Magnetic Resonance in Medicine / Society of Magnetic Resonance in Medicine, 60(3), 542–547.

Horská, A., & Barker, P. B. (2010). Imaging of brain tumors: MR spectroscopy and metabolic imaging. Neuroimaging Clinics of North America, 20(3), 293–310.

Lin, A., Andronesi, O., Bogner, W., Choi, I.-Y., Coello, E., Cudalbu, C., Juchem, C., Kemp, G. J., Kreis, R., Krššák, M., Lee, P., Maudsley, A. A., Meyerspeer, M., Mlynarik, V., Near, J., Öz, G., Peek, A. L., Puts, N. A., Ratai, E.-M., … Experts’ Working Group on Reporting Standards for MR Spectroscopy. (2021). Minimum Reporting Standards for in vivo Magnetic Resonance Spectroscopy (MRSinMRS): Experts’ consensus recommendations. NMR in Biomedicine, 34(5), e4484.

Maudsley, A. A., Andronesi, O. C., Barker, P. B., Bizzi, A., Bogner, W., Henning, A., Nelson, S. J., Posse, S., Shungu, D. C., & Soher, B. J. (2021). Advanced magnetic resonance spectroscopic neuroimaging: Experts’ consensus recommendations. NMR in Biomedicine, 34(5), e4309.

Oeltzschner, G. (2020, April 9). MRSHub. MRSHub. https://mrshub.org/

Öz, G., Deelchand, D. K., Wijnen, J. P., Mlynárik, V., Xin, L., Mekle, R., Noeske, R., Scheenen, T. W. J., Tkáč, I., & Experts’ Working Group on Advanced Single Voxel 1H MRS. (2020). Advanced single voxel 1 H magnetic resonance spectroscopy techniques in humans: Experts’ consensus recommendations. NMR in Biomedicine, e4236.

Ozturk-Isik, E., Cengiz, S., Ozcan, A., Yakicier, C., Ersen Danyeli, A., Pamir, M. N., Özduman, K., & Dincer, A. (2020). Identification of IDH and TERTp mutation status using 1 H-MRS in 112 hemispheric diffuse gliomas. Journal of Magnetic Resonance Imaging: JMRI, 51(6), 1799–1809.

Považan, M., Mikkelsen, M., Berrington, A., Bhattacharyya, P. K., Brix, M. K., Buur, P. F., Cecil, K. M., Chan, K. L., Chen, D. Y. T., Craven, A. R., Cuypers, K., Dacko, M., Duncan, N. W., Dydak, U., Edmondson, D. A., Ende, G., Ersland, L., Forbes, M. A., Gao, F., … Barker, P. B. (2020). Comparison of Multivendor Single-Voxel MR Spectroscopy Data Acquired in Healthy Brain at 26 Sites. Radiology, 295(1), 171–180.

Sabati, M., Sheriff, S., Gu, M., Wei, J., Zhu, H., Barker, P. B., Spielman, D. M., Alger, J. R., & Maudsley, A. A. (2015). Multivendor implementation and comparison of volumetric whole-brain echo-planar MR spectroscopic imaging. Magnetic Resonance in Medicine: Official Journal of the Society of Magnetic Resonance in Medicine / Society of Magnetic Resonance in Medicine, 74(5), 1209–1220.

Terpstra, M., Cheong, I., Lyu, T., Deelchand, D. K., Emir, U. E., Bednařík, P., Eberly, L. E., & Öz, G. (2016). Test-retest reproducibility of neurochemical profiles with short-echo, single-voxel MR spectroscopy at 3T and 7T. Magnetic Resonance in Medicine: Official Journal of the Society of Magnetic Resonance in Medicine / Society of Magnetic Resonance in Medicine, 76(4), 1083–1091.

Wang, Q., Zhang, H., Zhang, J., Wu, C., Zhu, W., Li, F., Chen, X., & Xu, B. (2016). The diagnostic performance of magnetic resonance spectroscopy in differentiating high-from low-grade gliomas: A systematic review and meta-analysis. European Radiology, 26(8), 2670–2684.

Wang, W., Hu, Y., Lu, P., Li, Y., Chen, Y., Tian, M., & Yu, L. (2014). Evaluation of the diagnostic performance of magnetic resonance spectroscopy in brain tumors: a systematic review and meta-analysis. PloS One, 9(11), e112577.

Wilson, M., Andronesi, O., Barker, P. B., Bartha, R., Bizzi, A., Bolan, P. J., Brindle, K. M., Choi, I.-Y., Cudalbu, C., Dydak, U., Emir, U. E., Gonzalez, R. G., Gruber, S., Gruetter, R., Gupta, R. K., Heerschap, A., Henning, A., Hetherington, H. P., Huppi, P. S., … Howe, F. A. (2019). Methodological consensus on clinical proton MRS of the brain: Review and recommendations. Magnetic Resonance in Medicine: Official Journal of the Society of Magnetic Resonance in Medicine / Society of Magnetic Resonance in Medicine, 82(2), 527–550.

## References for the Level of Validation Table - CEST

Herz, K., Mueller, S., Perlman, O., Zaitsev, M., Knutsson, L., Sun, P. Z., Zhou, J., van Zijl, P., Heinecke, K., Schuenke, P., Farrar, C. T., Schmidt, M., Dörfler, A., Scheffler, K., & Zaiss, M. (2021). Pulseq-CEST: Towards multi-site multi-vendor compatibility and reproducibility of CEST experiments using an open-source sequence standard. Magnetic Resonance in Medicine: Official Journal of the Society of Magnetic Resonance in Medicine / Society of Magnetic Resonance in Medicine, 86(4), 1845–1858.

Lingl, J. P., Wunderlich, A., Goerke, S., Paech, D., Ladd, M. E., Liebig, P., Pala, A., Kim, S. Y., Braun, M., Schmitz, B. L., Beer, M., & Rosskopf, J. (2022). The Value of APTw CEST MRI in Routine Clinical Assessment of Human Brain Tumor Patients at 3T. Diagnostics (Basel, Switzerland), 12(2). https://doi.org/10.3390/diagnostics12020490

Mancini, L., Casagranda, S., Gautier, G., Peter, P., Lopez, B., Thorne, L., McEvoy, A., Miserocchi, A., Samandouras, G., Kitchen, N., Brandner, S., De Vita, E., Torrealdea, F., Rega, M., Schmitt, B., Liebig, P., Sanverdi, E., Golay, X., & Bisdas, S. (2022). CEST MRI provides amide/amine surrogate biomarkers for treatment-naïve glioma sub-typing. European Journal of Nuclear Medicine and Molecular Imaging, 49(7), 2377–2391.

Suh, C. H., Park, J. E., Jung, S. C., Choi, C. G., Kim, S. J., & Kim, H. S. (2019). Amide proton transfer-weighted MRI in distinguishing high- and low-grade gliomas: a systematic review and meta-analysis. Neuroradiology, 61(5), 525–534.

Togao, O., Yoshiura, T., Keupp, J., Hiwatashi, A., Yamashita, K., Kikuchi, K., Suzuki, Y., Suzuki, S. O., Iwaki, T., Hata, N., Mizoguchi, M., Yoshimoto, K., Sagiyama, K., Takahashi, M., & Honda, H. (2014). Amide proton transfer imaging of adult diffuse gliomas: correlation with histopathological grades. Neuro-Oncology, 16(3), 441–448.

Wamelink, I. J. H. G., Kuijer, J. P. A., Padrela, B. E., Zhang, Y., Barkhof, F., Mutsaerts, H. J. M. M., Petr, J., van de Giessen, E., & Keil, V. C. (2022). Reproducibility of 3 T APT-CEST in Healthy Volunteers and Patients With Brain Glioma. Journal of Magnetic Resonance Imaging: JMRI. https://doi.org/10.1002/jmri.28239

Warnert, E. A. H., Wood, T. C., Incekara, F., Barker, G. J., Vincent, A. J. P., Schouten, J., Kros, J. M., van den Bent, M., Smits, M., & Tamames, J. A. H. (2022). Mapping tumour heterogeneity with pulsed 3D CEST MRI in non-enhancing glioma at 3 T. Magma, 35(1), 53–62.

Zhang, J., Zhu, W., Tain, R., Zhou, X. J., & Cai, K. (2018). Improved Differentiation of Low-Grade and High-Grade Gliomas and Detection of Tumor Proliferation Using APT Contrast Fitted from Z-Spectrum. Molecular Imaging and Biology: MIB: The Official Publication of the Academy of Molecular Imaging, 20(4), 623–631.

Zhou, J., Blakeley, J. O., Hua, J., Kim, M., Laterra, J., Pomper, M. G., & van Zijl, P. C. M. (2008). Practical data acquisition method for human brain tumor amide proton transfer (APT) imaging. Magnetic Resonance in Medicine: Official Journal of the Society of Magnetic Resonance in Medicine / Society of Magnetic Resonance in Medicine, 60(4), 842–849.

Zhou, J., Zaiss, M., Knutsson, L., Sun, P. Z., Ahn, S. S., Aime, S., Bachert, P., Blakeley, J. O., Cai, K., Chappell, M. A., Chen, M., Gochberg, D. F., Goerke, S., Heo, H.-Y., Jiang, S., Jin, T., Kim, S.-G., Laterra, J., Paech, D., … van Zijl, P. C. M. (2022). Review and consensus recommendations on clinical APT-weighted imaging approaches at 3T: Application to brain tumors. Magnetic Resonance in Medicine: Official Journal of the Society of Magnetic Resonance in Medicine / Society of Magnetic Resonance in Medicine, 88(2), 546–574.

## References for the Level of Validation Table - SWI

Baeshen, A., Wyss, P. O., Henning, A., O’Gorman, R. L., Piccirelli, M., Kollias, S., & Michels, L. (2020). Test-Retest Reliability of the Brain Metabolites GABA and Glx With JPRESS, PRESS, and MEGA-PRESS MRS Sequences in vivo at 3T. Journal of Magnetic Resonance Imaging: JMRI, 51(4), 1181–1191.

Ding, J., Duan, Y., Wang, M., Yuan, Y., Zhuo, Z., Gan, L., Song, Q., Gao, B., Yang, L., Liu, H., Hou, Y., Zheng, F., Chen, R., Wang, J., Lin, L., Zhang, B., Zhang, G., & Liu, Y. (2022). Acceleration of Brain Susceptibility-Weighted Imaging with Compressed Sensitivity Encoding: A Prospective Multicenter Study. AJNR. American Journal of Neuroradiology, 43(3), 402–409.

Haacke, E. M., Mittal, S., Wu, Z., Neelavalli, J., & Cheng, Y.-C. N. (2009). Susceptibility-weighted imaging: technical aspects and clinical applications, part 1. AJNR. American Journal of Neuroradiology, 30(1), 19–30.

Hsu, C. C.-T., Watkins, T. W., Kwan, G. N. C., & Haacke, E. M. (2016). Susceptibility-Weighted Imaging of Glioma: Update on Current Imaging Status and Future Directions. Journal of Neuroimaging: Official Journal of the American Society of Neuroimaging, 26(4), 383–390.

Lancione, M., Bosco, P., Costagli, M., Nigri, A., Aquino, D., Carne, I., Ferraro, S., Giulietti, G., Napolitano, A., Palesi, F., Pavone, L., Pirastru, A., Savini, G., Tagliavini, F., Bruzzone, M. G., Gandini Wheeler-Kingshott, C. A. M., Tosetti, M., Biagi, L., & RIN – Neuroimaging Network. (2022). Multi-centre and multi-vendor reproducibility of a standardized protocol for quantitative susceptibility Mapping of the human brain at 3T. Physica Medica: PM: An International Journal Devoted to the Applications of Physics to Medicine and Biology: Official Journal of the Italian Association of Biomedical Physics , 103, 37–45.

Reichenbach, J. R., Schweser, F., Serres, B., & Deistung, A. (2015). Quantitative Susceptibility Mapping: Concepts and Applications. Clinical Neuroradiology, 25 Suppl 2, 225–230.

Rua, C., Clarke, W. T., Driver, I. D., Mougin, O., Morgan, A. T., Clare, S., Francis, S., Muir, K. W., Wise, R. G., Carpenter, T. A., Williams, G. B., Rowe, J. B., Bowtell, R., & Rodgers, C. T. (2020). Multi-centre, multi-vendor reproducibility of 7T QSM and R2* in the human brain: Results from the UK7T study. NeuroImage, 223, 117358.

Wu, Y., Den, Z., & Lin, Y. (2018). Accuracy of Susceptibility-Weighted Imaging and Dynamic Susceptibility Contrast Magnetic Resonance Imaging for Differentiating High-Grade Glioma from Primary Central Nervous System Lymphomas: Meta-Analysis. World Neurosurgery, 112, e617–e623.

## References for the Level of Validation Table - MRE

Bunevicius, A., Schregel, K., Sinkus, R., Golby, A., & Patz, S. (2020). REVIEW: MR elastography of brain tumors. NeuroImage. Clinical, 25, 102109.

Dickerson, E., & Srinivasan, A. (2016). Multicenter Survey of Current Practice Patterns in Perfusion MRI in Neuroradiology: Why, When, and How Is It Performed? AJR. American Journal of Roentgenology, 207(2), 406–410.

Ellingson, B. M., Bendszus, M., Boxerman, J., Barboriak, D., Erickson, B. J., Smits, M., Nelson, S. J., Gerstner, E., Alexander, B., Goldmacher, G., Wick, W., Vogelbaum, M., Weller, M., Galanis, E., Kalpathy-Cramer, J., Shankar, L., Jacobs, P., Pope, W. B., Yang, D., … Jumpstarting Brain Tumor Drug Development Coalition Imaging Standardization Steering Committee. (2015). Consensus recommendations for a standardized Brain Tumor Imaging Protocol in clinical trials. Neuro-Oncology, 17(9), 1188–1198.

Ellingson, B. M., Wen, P. Y., & Cloughesy, T. F. (2017). Modified Criteria for Radiographic Response Assessment in Glioblastoma Clinical Trials. Neurotherapeutics: The Journal of the American Society for Experimental NeuroTherapeutics, 14(2), 307–320.

Fløgstad Svensson, S., Fuster-Garcia, E., Latysheva, A., Fraser-Green, J., Nordhøy, W., Isam Darwish, O., Thokle Hovden, I., Holm, S., Vik-Mo, E. O., Sinkus, R., & Eeg Emblem, K. (2022). Decreased tissue stiffness in glioblastoma by MR elastography is associated with increased cerebral blood flow. European Journal of Radiology, 147, 110136.

Johnson, C. L., McGarry, M. D. J., Gharibans, A. A., Weaver, J. B., Paulsen, K. D., Wang, H., Olivero, W. C., Sutton, B. P., & Georgiadis, J. G. (2013). Local mechanical properties of white matter structures in the human brain. NeuroImage, 79, 145–152.

Johnson, C. L., Schwarb, H., D J McGarry, M., Anderson, A. T., Huesmann, G. R., Sutton, B. P., & Cohen, N. J. (2016). Viscoelasticity of subcortical gray matter structures. Human Brain Mapping, 37(12), 4221–4233.

Manduca, A., Bayly, P. J., Ehman, R. L., Kolipaka, A., Royston, T. J., Sack, I., Sinkus, R., & Van Beers, B. E. (2021). MR elastography: Principles, guidelines, and terminology. Magnetic Resonance in Medicine: Official Journal of the Society of Magnetic Resonance in Medicine / Society of Magnetic Resonance in Medicine, 85(5), 2377–2390.

McGarry, M., Johnson, C. L., Sutton, B. P., Van Houten, E. E., Georgiadis, J. G., Weaver, J. B., & Paulsen, K. D. (2013). Including spatial information in nonlinear inversion MR elastography using soft prior regularization. IEEE Transactions on Medical Imaging, 32(10), 1901–1909.

Meyer, T., Marticorena Garcia, S., Tzschätzsch, H., Herthum, H., Shahryari, M., Stencel, L., Braun, J., Kalra, P., Kolipaka, A., & Sack, I. (2022). Comparison of inversion methods in MR elastography: An open-access pipeline for processing multifrequency shear-wave data and demonstration in a phantom, human kidneys, and brain. Magnetic Resonance in Medicine: Official Journal of the Society of Magnetic Resonance in Medicine / Society of Magnetic Resonance in Medicine, 88(4), 1840–1850.

Murphy, M. C., Huston, J., 3rd, Jack, C. R., Jr, Glaser, K. J., Senjem, M. L., Chen, J., Manduca, A., Felmlee, J. P., & Ehman, R. L. (2013). Measuring the characteristic topography of brain stiffness with magnetic resonance elastography. PloS One, 8(12), e81668.

O’Connor, J. P. B., Aboagye, E. O., Adams, J. E., Aerts, H. J. W. L., Barrington, S. F., Beer, A. J., Boellaard, R., Bohndiek, S. E., Brady, M., Brown, G., Buckley, D. L., Chenevert, T. L., Clarke, L. P., Collette, S., Cook, G. J., deSouza, N. M., Dickson, J. C., Dive, C., Evelhoch, J. L., … Waterton, J. C. (2017). Imaging biomarker roadmap for cancer studies. Nature Reviews. Clinical Oncology, 14(3), 169–186.

Pepin, K. M., McGee, K. P., Arani, A., Lake, D. S., Glaser, K. J., Manduca, A., Parney, I. F., Ehman, R. L., & Huston, J., 3rd. (2018). MR Elastography Analysis of Glioma Stiffness and IDH1-Mutation Status. AJNR. American Journal of Neuroradiology, 39(1), 31–36.

Reiss-Zimmermann, M., Streitberger, K.-J., Sack, I., Braun, J., Arlt, F., Fritzsch, D., & Hoffmann, K.-T. (2015). High Resolution Imaging of Viscoelastic Properties of Intracranial Tumours by Multi-Frequency Magnetic Resonance Elastography. Clinical Neuroradiology, 25(4), 371–378.

Streitberger, K.-J., Reiss-Zimmermann, M., Freimann, F. B., Bayerl, S., Guo, J., Arlt, F., Wuerfel, J., Braun, J., Hoffmann, K.-T., & Sack, I. (2014). High-resolution mechanical imaging of glioblastoma by multifrequency magnetic resonance elastography. PloS One, 9(10), e110588.

Svensson, S. F., De Arcos, J., Darwish, O. I., Fraser-Green, J., Storås, T. H., Holm, S., Vik-Mo, E. O., Sinkus, R., & Emblem, K. E. (2021). Robustness of MR Elastography in the Healthy Brain: Repeatability, Reliability, and Effect of Different Reconstruction Methods. Journal of Magnetic Resonance Imaging: JMRI, 53(5), 1510–1521.

Thust, S. C., Heiland, S., Falini, A., Jäger, H. R., Waldman, A. D., Sundgren, P. C., Godi, C., Katsaros, V. K., Ramos, A., Bargallo, N., Vernooij, M. W., Yousry, T., Bendszus, M., & Smits, M. (2018). Glioma imaging in Europe: A survey of 220 centres and recommendations for best clinical practice. European Radiology, 28(8), 3306–3317.

Trout, A. T., Serai, S., Mahley, A. D., Wang, H., Zhang, Y., Zhang, B., & Dillman, J. R. (2016). Liver Stiffness Measurements with MR Elastography: Agreement and Repeatability across Imaging Systems, Field Strengths, and Pulse Sequences. Radiology, 281(3), 793–804.

Weller, M., van den Bent, M., Preusser, M., Le Rhun, E., Tonn, J. C., Minniti, G., Bendszus, M., Balana, C., Chinot, O., Dirven, L., French, P., Hegi, M. E., Jakola, A. S., Platten, M., Roth, P., Rudà, R., Short, S., Smits, M., Taphoorn, M. J. B., … Wick, W. (2021). EANO guidelines on the diagnosis and treatment of diffuse gliomas of adulthood. Nature Reviews. Clinical Oncology, 18(3), 170–186.

Weller, M., van den Bent, M., Tonn, J. C., Stupp, R., Preusser, M., Cohen-Jonathan-Moyal, E., Henriksson, R., Le Rhun, E., Balana, C., Chinot, O., Bendszus, M., Reijneveld, J. C., Dhermain, F., French, P., Marosi, C., Watts, C., Oberg, I., Pilkington, G., Baumert, B. G., … European Association for Neuro-Oncology (EANO) Task Force on Gliomas. (2017). European Association for Neuro-Oncology (EANO) guideline on the diagnosis and treatment of adult astrocytic and oligodendroglial gliomas. The Lancet Oncology, 18(6), e315–e329.

Wen, P. Y., Chang, S. M., Van den Bent, M. J., Vogelbaum, M. A., Macdonald, D. R., & Lee, E. Q. (2017). Response Assessment in Neuro-Oncology Clinical Trials. Journal of Clinical Oncology: Official Journal of the American Society of Clinical Oncology, 35(21), 2439–2449.

Wen, P. Y., Weller, M., Lee, E. Q., Alexander, B. M., Barnholtz-Sloan, J. S., Barthel, F. P., Batchelor, T. T., Bindra, R. S., Chang, S. M., Chiocca, E. A., Cloughesy, T. F., DeGroot, J. F., Galanis, E., Gilbert, M. R., Hegi, M. E., Horbinski, C., Huang, R. Y., Lassman, A. B., Le Rhun, E., … van den Bent, M. J. (2020). Glioblastoma in adults: a Society for Neuro-Oncology (SNO) and European Society of Neuro-Oncology (EANO) consensus review on current management and future directions. Neuro-Oncology, 22(8), 1073–1113.

## References for the Level of Validation Table - MR-PET

Katsanos, A. H., Alexiou, G. A., Fotopoulos, A. D., Jabbour, P., Kyritsis, A. P., & Sioka, C. (2019). Performance of 18F-FDG, 11C-Methionine, and 18F-FET PET for Glioma Grading: A Meta-analysis. Clinical Nuclear Medicine, 44(11), 864–869.

Law, I., Albert, N. L., Arbizu, J., Boellaard, R., Drzezga, A., Galldiks, N., la Fougère, C., Langen, K.-J., Lopci, E., Lowe, V., McConathy, J., Quick, H. H., Sattler, B., Schuster, D. M., Tonn, J.-C., & Weller, M. (2019). Joint EANM/EANO/RANO practice guidelines/SNMMI procedure standards for imaging of gliomas using PET with radiolabelled amino acids and [18F]FDG: version 1.0. European Journal of Nuclear Medicine and Molecular Imaging, 46(3), 540–557.

Lodge, M. A., Holdhoff, M., Leal, J. P., Bag, A. K., Nabors, L. B., Mintz, A., Lesser, G. J., Mankoff, D. A., Desai, A. S., Mountz, J. M., Lieberman, F. S., Fisher, J. D., Desideri, S., Ye, X., Grossman, S. A., Schiff, D., & Wahl, R. L. (2017). Repeatability of 18F-FLT PET in a Multicenter Study of Patients with High-Grade Glioma. Journal of Nuclear Medicine: Official Publication, Society of Nuclear Medicine, 58(3), 393–398.

## References for the Level of Validation Table - Guidelines

- a Imaging biomarker roadmap (O’Connor et al., 2017)
- b RANO (Wen et al., 2017), iRANO (Ellingson et al., 2017), Standardised Brain Tumor Imaging Protocol (Ellingson et al., 2015)
- c GBM EANO/SNO (Wen et al., 2020), EANO diff. glioma (Weller et al., 2021), EANO glioma (Weller et al., 2017)
- d European survey of adv.MRI (Thust et al., 2018), US survey perfusion imaging (Dickerson & Srinivasan, 2016).

Dickerson, E., & Srinivasan, A. (2016). Multicenter Survey of Current Practice Patterns in Perfusion MRI in Neuroradiology: Why, When, and How Is It Performed? AJR. American Journal of Roentgenology, 207(2), 406–410.

Ellingson, B. M., Bendszus, M., Boxerman, J., Barboriak, D., Erickson, B. J., Smits, M., Nelson, S. J., Gerstner, E., Alexander, B., Goldmacher, G., Wick, W., Vogelbaum, M., Weller, M., Galanis, E., Kalpathy-Cramer, J., Shankar, L., Jacobs, P., Pope, W. B., Yang, D., … Jumpstarting Brain Tumor Drug Development Coalition Imaging Standardization Steering Committee. (2015). Consensus recommendations for a standardized Brain Tumor Imaging Protocol in clinical trials. Neuro-Oncology, 17(9), 1188–1198.

Ellingson, B. M., Wen, P. Y., & Cloughesy, T. F. (2017). Modified Criteria for Radiographic Response Assessment in Glioblastoma Clinical Trials. Neurotherapeutics: The Journal of the American Society for Experimental NeuroTherapeutics, 14(2), 307–320.

O’Connor, J. P. B., Aboagye, E. O., Adams, J. E., Aerts, H. J. W. L., Barrington, S. F., Beer, A. J., Boellaard, R., Bohndiek, S. E., Brady, M., Brown, G., Buckley, D. L., Chenevert, T. L., Clarke, L. P., Collette, S., Cook, G. J., deSouza, N. M., Dickson, J. C., Dive, C., Evelhoch, J. L., … Waterton, J. C. (2017). Imaging biomarker roadmap for cancer studies. Nature Reviews. Clinical Oncology, 14(3), 169–186.

Thust, S. C., Heiland, S., Falini, A., Jäger, H. R., Waldman, A. D., Sundgren, P. C., Godi, C., Katsaros, V. K., Ramos, A., Bargallo, N., Vernooij, M. W., Yousry, T., Bendszus, M., & Smits, M. (2018). Glioma imaging in Europe: A survey of 220 centres and recommendations for best clinical practice. European Radiology, 28(8), 3306–3317.

Weller, M., van den Bent, M., Preusser, M., Le Rhun, E., Tonn, J. C., Minniti, G., Bendszus, M., Balana, C., Chinot, O., Dirven, L., French, P., Hegi, M. E., Jakola, A. S., Platten, M., Roth, P., Rudà, R., Short, S., Smits, M., Taphoorn, M. J. B., … Wick, W. (2021). EANO guidelines on the diagnosis and treatment of diffuse gliomas of adulthood. Nature Reviews. Clinical Oncology, 18(3), 170–186.

Weller, M., van den Bent, M., Tonn, J. C., Stupp, R., Preusser, M., Cohen-Jonathan-Moyal, E., Henriksson, R., Le Rhun, E., Balana, C., Chinot, O., Bendszus, M., Reijneveld, J. C., Dhermain, F., French, P., Marosi, C., Watts, C., Oberg, I., Pilkington, G., Baumert, B. G., … European Association for Neuro-Oncology (EANO) Task Force on Gliomas. (2017). European Association for Neuro-Oncology (EANO) guideline on the diagnosis and treatment of adult astrocytic and oligodendroglial gliomas. The Lancet Oncology, 18(6), e315–e329.

Wen, P. Y., Chang, S. M., Van den Bent, M. J., Vogelbaum, M. A., Macdonald, D. R., & Lee, E. Q. (2017). Response Assessment in Neuro-Oncology Clinical Trials. Journal of Clinical Oncology: Official Journal of the American Society of Clinical Oncology, 35(21), 2439–2449.

Wen, P. Y., Weller, M., Lee, E. Q., Alexander, B. M., Barnholtz-Sloan, J. S., Barthel, F. P., Batchelor, T. T., Bindra, R. S., Chang, S. M., Chiocca, E. A., Cloughesy, T. F., DeGroot, J. F., Galanis, E., Gilbert, M. R., Hegi, M. E., Horbinski, C., Huang, R. Y., Lassman, A. B., Le Rhun, E., … van den Bent, M. J. (2020). Glioblastoma in adults: a Society for Neuro-Oncology (SNO) and European Society of Neuro-Oncology (EANO) consensus review on current management and future directions. Neuro-Oncology, 22(8), 1073–1113.
